# Cytosolic- and membrane-localized oxidized indole-3-acetic acid formation regulates developmental auxin transients

**DOI:** 10.1093/plphys/kiaf330

**Published:** 2025-08-04

**Authors:** Mark Jenness, Reuben Tayengwa, Lillyanna House, Shiyun Cao, Román Ramos Báez, Seema Nath, Candace A. Pritchard, Anket Sharma, Julia Mergner, Frédèric Rivière, Thierry Meinnel, Carmela Giglione, Bernhard Kuster, Joshua J. Blakeslee, Jennifer L. Nemhauser, Ning Zheng, Angus S. Murphy, Wendy Ann Peer

**Affiliations:** a Department of Pant Science and Landscape Architecture, University of Maryland, College Park, MD 20742; b Howard Hughes Medical Institute, Department of Pharmacology, University of Washington, Seattle, WA 98195, USA; c Department of Biology, University of Washington, Seattle, WA 98195, USA; d Department of Horticulture and Crop Science, Laboratory for the Analysis of Metabolites from Plants metabolomics facility, The Ohio State University, Columbus, OH, USA; e Proteomics and Bioanalytics, Technical University of Munich (TUM), Freising, Germany; Bavarian Center for Biomolecular Mass Spectrometry (BayBioMS), Technical University of Munich (TUM), Freising, Germany; f Université Paris-Saclay, CEA, CNRS, Institute for Integrative Biology of the Cell (I2BC), 91198 Gif-sur-Yvette, France; g Department of Environmental Science and Technology, University of Maryland, College Park, MD 20742; h Institute for Bioscience and Biotechnology Research, University of Maryland, College Park, MD 20742

**Keywords:** Auxin, oxIAA, apical hook, auxin signaling, DAO, IAA7, IAA17

## Abstract

Catabolism of the auxin indole-3-acetic acid (IAA) to terminate cellular responses primarily occurs in three steps: 1) conjugation of IAA to Asp/Glu, 2) oxidation of the indole ring by DIOXYENASE FOR AUXIN OXIDATION (DAO), and 3) amidohydrolase cleavage of Asp/Glu. This study examines if IAA oxidation historically associated with membranes is mediated by DAO isoforms and if oxidized auxin product (oxIAA) retains nominal functionality. We show that Arabidopsis DAO1 does exhibit both soluble and auxin-dependent plasma membrane association and that oxIAA exhibits weak “anti-auxin” activity. Both soluble and membrane-associated DAO1 primarily oxidize IAAsp. DAO2 activity is enzymatically similar to DAO1 and occurs where IAA levels are high. DAO1 and DAO2 function synergistically in adventitious root formation and in temperature-dependent petal development. *In vitro* assays show that oxIAA acts as a molecular glue between repressor AUX/IAA (IAA7 and IAA17) and TIR1 auxin co-receptors, but is readily outcompeted by IAA. BioLayer Interferometry and yeast degradation assays indicate weak “anti-auxin” activity, as oxIAA enhances IAA7-TIR1 interactions while retarding IAA7 and IAA17 degradation. In a low auxin/quiescent interval that precedes auxin-triggered apical hook opening in etiolated seedlings, IAA7 gain- and loss-of-function mutants exhibited early apical hook opening similar to *dao1*, and application of oxIAA to etiolated *dao1* apical hooks partially rescued the phenotype. The weak “anti-auxin” activity of oxIAA during a transitional growth is an important reminder of the evolutionary processes that forge adaptive plant growth responses.

## Introduction

The phytohormone auxin, primarily indole-3-actic acid (IAA), regulates almost every aspect of plant growth. Polarized auxin gradients generated by cell-to-cell transport maintain programmed and environmentally-responsive development. Cellular auxin homeostasis is rigorously maintained via regulation of auxin synthesis, metabolism, and compartmentalization. The primary post-synthetic cellular mechanism controlling cellular auxin levels is the conjugation of IAA to amino acids via the Gretchen Hagen 3 (GH3) acyl acid amido synthetases (Staswick et al., 2005; Ludwig-Muller, 2011). IAA conjugated to Phe, Leu, or Ala can serve as a storage form to be released and reactivated by amidohydrolases (Bartel and Fink, 1995; LeClere et al, 2002; Sanchez Carranza et al., 2016). When IAA is conjugated to Glu or Asp, the indole ring is preferentially oxidized by DIOXYGENASE FOR AUXIN OXIDATION (DAO) oxoglutarate-dependent oxidases to form 2-oxindole-3-acetic acyl (oxIA)Asp and oxIAGlu (Hayashi et al., 2021; Müller et al., 2021). Glu and Asp are then removed by cytosolic amidohydrolases to form the very weak auxin 2-oxindole-3-acetic (oxIAA) (Galston and Chen, 1965; Pěnčík et al., 2013; Hayashi et al., 2021). oxIAA levels are typically at least 5–10X greater than IAA in intact plant tissues and often change with IAA abundance (Pěnčík et al., 2013; Voß et al., 2015; Zheng et al., 2016). Cytosolic glycosyl transferases slowly conjugate glucose (Gluc) to oxIAA, and the conjugate is transported to the vacuole (Ranocha et al., 2013), where it is broken down. oxIAA is not polarly transported (Peer et al., 2013), but does appear to be inefficiently taken up when experimentally applied at the root apex (Kubeš, et al., 2012). The very slow rate of IAA oxidation by DAO and accumulation of IAGlu and IAAsp in Arabidopsis null *dao1* and *dao1 dao2* mutants (Hayashi et al., 2021) confirms that direct IAA oxidation is a minor component of auxin catabolism. However, twentieth-century reports of membrane-associated auxin oxidase activity (Waldrum and Davies, 1981; Reinecke and Bandurski, 1988) suggest that direct IAA oxidation could be relevant at these boundaries. A previous report in Arabidopsis (Zhang et al., 2016) visualized uniform DAO1 distribution in the cell, but biochemically identified DAO only in soluble fractions.

While almost all vascular plants harbor a single *DAO* gene (*DAO1*), in the Brassicaceae lineage, a gene duplication event gave rise to a second copy, *DAO2* (Zhang et al., 2016; Porco et al., 2016; Zhang and Peer, 2017). This diversification suggests an evolutionary adaptation to modulate auxin levels more precisely, a hypothesis supported by the identification of a putative DAO2 ortholog in rice (Takehara et al., 2020) and molecular genetic analyses in Arabidopsis (Voß et al., 2015; Wang et al., 2023) indicating the role of DAO2 in normal developmental processes under elevated auxin conditions.

Some oxidized auxins, including oxIAA, have been found to act as weak auxins or “anti-auxins” (Thimann, 1958). When oxIAA was applied to green pea stems, a “slight promotory activity” was observed (Galston and Chen, 1965). However, the observed elongation required 10X more oxIAA compared to 2,4-D to promote growth. Exogenous application of 1 μM oxIAA to Arabidopsis seedlings was previously shown to require 24 h before producing a visible increase in DR5rev:GFP signals in Arabidopsis roots (Pěnčík et al., 2013) and to have no visible impact on auxin reporter signals before that time (Peer et al., 2013). Pěnčík et al. (2013) showed that IAA7 can interact with TIR1 in the presence of oxIAA *in vitro*, but detailed analysis and physiological relevance is unknown.

Here we show that DAO1 is readily detectable in Arabidopsis membrane fractions in an auxin-dependent manner, and the results suggest that membrane-associated DAO1 may be slightly more active against IAA compared to soluble DAO1, but DAO1 primary oxidative activity is for IAAsp. We also show that DAO2 oxidation of IAA and IAAsp is also somewhat lower than DAO1, and that DAO2 developmental function is more evident in floral meristems and roots under environmental conditions where auxin levels are elevated. These results presented here also suggest a limited functional role of oxIAA as an anti-auxin in plant development that is consistent with observed *dao* mutant phenotypes, the presence of oxIAA as a late-stage catabolic intermediate before final glucosylation and transport into the vacuole, and the likelihood of unrecognized analog factors functioning in transitional developmental process when auxin levels decrease.

## Results

### Membrane and Soluble Auxin Oxidase Activities

#### Qualification of new dao2 and dao1 dao2 mutants

Assessments of DAO activity have primarily been performed in null *dao1-1* or knockdown *dao2-1* alleles (Zhang et al., 2016). Previously, *dao1-1* whole seedlings were shown to have reduced oxIAA levels (~5–10% of wild type) and elevated levels of IAAsp and IAGlu, but wild type IAA levels (Zhang et al., 2016; Porco et al., 2016; Hayashi et al., 2021; Müller et al., 2021), suggesting that *DAO2* activity might account for the residual oxIAA in the *dao1-1* null mutant. CRISPR-Cas9 was used to generate new null *dao2* single and *dao1 dao2* double mutants (Fig. S1A,B). Quantitative real-time PCR (qRT-PCR) showed that *DAO1* was not up-regulated in *dao2-2* or *dao2-3* nor detectable in either *dao1 dao2* double mutants, and that *DAO2* missense mRNAs were transcribed at low levels (Fig. S1C).

#### DAO1 is associated with soluble and membrane fractions

Both soluble and membrane-associated auxin oxidase activities have been described (Reinecke and Bandurski, 1983; Waldrum and David, 1981), and previous visualizations of both N- and C-terminal DAO fusions to fluorescent proteins in BY-2 and Arabidopsis (but not rice protoplasts) showed distinct cellular localization patterns (Zhao et al., 2013; Zhang et al., 2016; Porco et al., 2016; Müller et al., 2021; Takehara et al., 2020). Western blot analysis in those reports detected primarily cytosolic YFP-DAO1, but membrane preparations did utilize a high EDTA/ high pH buffer that has been shown to disrupt membrane association of peripheral membrane proteins (Engström and Zetterberg, 1981).

**Figure 1.**
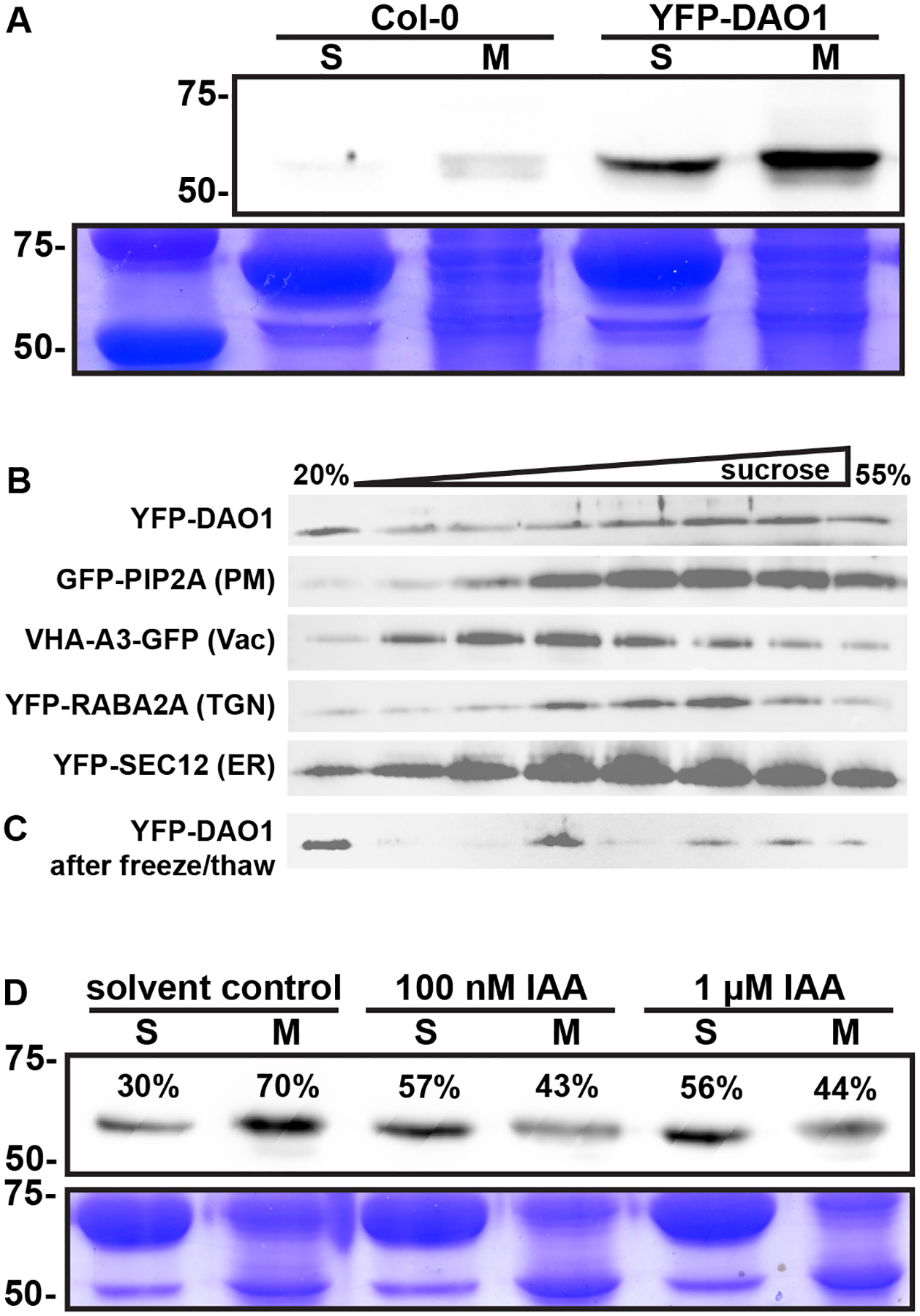
DAO1 is resident in soluble and membrane fractions. **(A)** Western blot analysis of YFP-DAO1 localization. S, soluble fraction; M, microsomal fraction. Position of 75 and 50 kDa ladder bands are indicated. **(B)** Microsomal membranes were separated on a continuous 20–55% sucrose density gradient, followed by SDS-PAGE and Western blotting. GFP-PIP2A, VHA-A3-GFP, YFP-RABA2A, and YFP-SEC12 were used as markers for the plasma membrane (PM), vacuole (Vac), *trans*-Golgi network (TGN), and endoplasmic reticulum (ER), respectively. **(C)** YFP-DAO1 after freeze/thaw. **(D)** Western blot analysis of YFP-DAO1 localization after 100 nM or 1 mM auxin treatment. S, soluble fraction; M, microsomal fraction. Position of 75 and 50 kDa ladder bands are indicated. The values were determined by densitometry.

To better distinguish membrane and cytosolic DAO abundance, soluble and microsomal proteins were isolated from Col-0 and *DAO1*:YFP-DAO1 and *DAO2*:YFP-DAO2 seedlings using 5 mM EDTA at pH 7.5 (Yang et al., 2013). SDS-PAGE and western blot analysis revealed a band of the expected size (~63 kDa) in isolations from the *DAO1*:YFP-DAO1 and *DAO2*:YFP-DAO2 lines but not Col-0 (Fig. 1A; Fig. S2), although faint, non-specific bands were observed in controls as well. Under these conditions, YFP-DAO1 and YFP-DAO2 were associated with both the soluble and membrane fractions, consistent with reported confocal microscopic localizations and auxin oxidase activity in both soluble and membrane fractions.

#### Membrane-associated DAO1 is primarily found in plasma membrane fractions

Western blots of sucrose density fractions with YFP antisera detected YFP-DAO1 in multiple fractions (Fig. 1B) most prominently overlapping with GFP-PIP2A and secondarily with the endoplasmic reticulum marker YFP-SEC12 (Fig. 1B). Membrane association of peripheral membrane proteins and proteins with weak or transient membrane interactions can be disrupted by freeze/thaw cycles. After one freeze/thaw cycle, YFP-DAO1 signals largely shifted to the top of the sucrose density gradient (Fig. 1C), a fraction that is indicative of free soluble protein. Efforts to further identify plasma membrane DAO activity by PEG-Dextran phase separation (Yang et al., 2013) resulted in loss of DAO1 from the membrane, consistent with a relatively weak association.

#### DAO1 membrane association is reduced by auxin treatment

In cell fractions, the majority (70%) of YFP-DAO1 was associated with the membrane fraction (Fig. 1A, D). After treatment with 100 nM or 1 μM IAA, membrane-associated DAO1 decreased to about 40% (Fig. 1D; Table S1). These combined data suggest that DAO1 exhibits membrane and cytosolic localizations in specific tissues in response to local auxin concentration levels.

DAO1 crystal structures and modeling (Jin et al., 2020; Takehara et al., 2020) show that under low auxin conditions, DAO1 is observed in both monomeric and dimeric forms with auxin treatment. Diagnostic elution profiles confirmed that dimeric DAO1 increased with auxin treatment as described previously, and bimolecular florescence assays showed cytosolic localization (Takehara et al., 2020; Hayashi et al., 2021; Müller et al., 2021). Under low IAA levels, DAO1 appears to exist primarily as a monomer with increased IAA coinciding with an increase in dimeric DAO1 (Takehara et al., 2020). Here, increased IAA decreases DAO1 plasma membrane association (Fig. 1), suggesting DAO1 may reside on the plasma membrane as a monomer.

Zhang et al. (2016) suggested that reversible co- and post-translation modifications such as acetylation, myristylation, and phosphorylation might modify DAO membrane association, as amino acid motifs consistent with all three types of modification are found in DAO1. DAO1 displays an N-terminal (Nt) glycine that can undergo modification. Nt-acetylation of DAO has been detected by proteomic analyses (Mergner et al., 2020), and the DAO1 protein N-terminus undergoes cleavage of the initiator methionine followed by acetylation of the exposed glycine residue (Fig. S3A). However, *in vitro* assays did not detect myristoylation (Myr) at any enzyme concentration (Fig. S3B).

Phosphorylation could also impact DAO residence on the membrane as is observed with phot1 with blue light (Kaiserli et al., 2019), as massive changes in phosphorylation status are evident with auxin treatment (Kuhn et al., 2024). However, phosphorylation at key amino acid residues could not be detected in DAO1 or DAO2 (Fig. S3C), although the peptide sequence coverage of DAO1 and DAO2 in phosphoproteomics was 48% and 27%, respectively.

#### Auxin oxidase activity in soluble and membrane fractions

Previously described oxidation assays measured via HPLC (Hayashi et al., 2021; Müller et al., 2021) were replicated with recombinant DAO1 and DAO2 with results assayed by LC-MS. They confirmed that enzymatic oxidation of IAAsp is a minimum of 10X greater than minimal oxidation of IAA (Table S2, Table S3). In an effort to compare recombinant and native activities, Col-0 soluble fractions were incubated with IAA and Mg-ATP. After 30 min, oxIAA and a small amount of IAAsp and oxIAAsp were detected (Table S4A). Reaction products in *dao2* resembled Col-0, and all IAA oxidation products were greatly reduced in *dao1dao2*. In the *ilr1ill2iar3* triple amidohydrolase mutant, IAAsp and oxIAAsp were elevated, consistent with a primary IAA > IAAsp > oxIAAsp > oxIAA catabolism pathway (Hayashi et al., 2021). Small amounts of persistent endogenous IAA metabolites in prepared homogenates were observed in solvent controls as would be expected (Table S4B,C).

The possibility that membrane-resident DAO might exhibit increased affinity for IAA was investigated. Incubations (30 min) of IAA with Col-0 membrane fractions contained small amounts of oxIAA (Table S4A) indicative of some direct oxidation, as soluble GH3 enzymes are absent in membrane fractions. Residual oxidative activity evident in *dao1* fractions suggested lesser DAO2 membrane function. As a final step, soluble fractions from *ilr1ill2iar3* were combined with microsomal membranes from *dao1* in an “add back” assay (Table S4D). Small amounts of IAAsp and oxIAAsp, but not oxIAA were detected in the reaction after addition of IAA, but only oxIAAsp was detected after addition of IAAsp. These assays demonstrated a preferential oxidation of IAAsp by both cytosolic and membrane-resident DAO with only a minor level of direct IAA oxidation in membrane fractions.

### Contributions of DAO2 activity to *dao1* phenotypes

#### Auxin metabolites accumulate differentially in null dao lines

**Figure 2.**
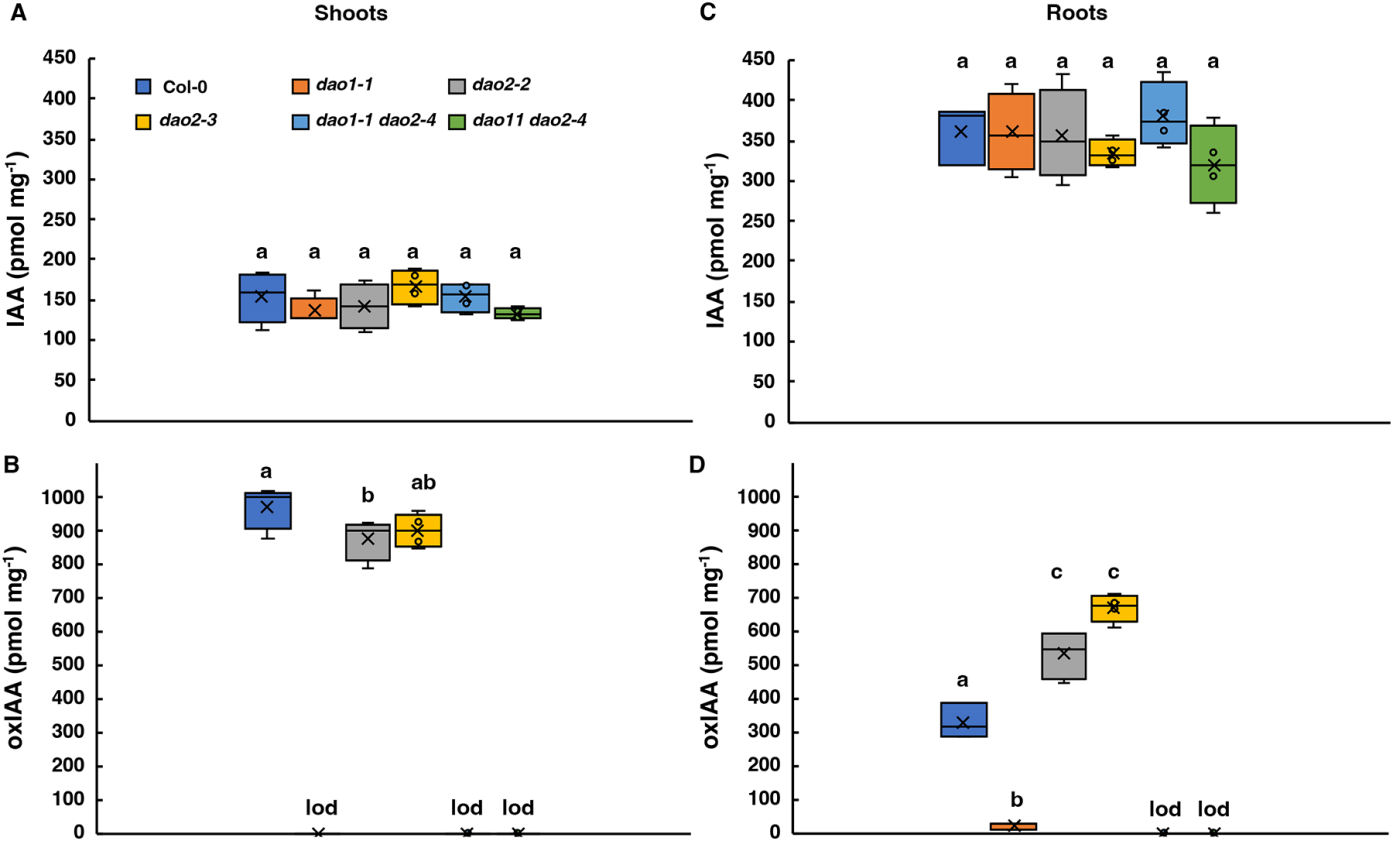
oxIAA metabolite levels in light-grown *dao1*, *dao2*, and *dao1 dao2* mutants support DAO1 is the primary oxidase. IAA (**A, C**) and oxIAA (**B, D**) levels in 7 d seedlings grown in continuous light **(A, B)** shoots, **(B, C)** roots. n = 5 biological replicates. Letters indicate statistical differences by ANOVA Holm-Sidak’s multiple comparisons test (P < 0.05). “lod”, limit of detection.

In light-grown 7 d *dao1 dao2* seedlings separated into roots and shoots, oxIAA was below the limit of detection, and free auxin and auxin metabolite levels were similar to *dao1-1* (Fig. 2; Fig. S4). Single *dao2* mutant roots and shoots also contained wild type levels of free IAA (Fig. 2A, C), but oxIAA levels in *dao2* roots were greater than wild type (P < 0.05; Fig. 2D). A lack of compensatory *DAO1* expression in *dao2* mutants (Fig. S1C) resulted wild type levels of IAAsp and IAGlu (P > 0.05; Fig. S4), while *GH3.3* expression consistent with wild type, but not *dao1* (P > 0.05; Fig. S1C), and suggests DAO1 post-translational modification and/or modified indolic precursor metabolism in *dao2*. A notably consistent feature of dark-grown roots is that IAAsp and IAGlu were at or near the limit of detection (Fig. S4C, D).

### DAO1 and DAO2 expression patterns and loss-of-function phenotypes

#### DAO1 and DAO2 display tissue-specific expression patterns

**Figure 3.**
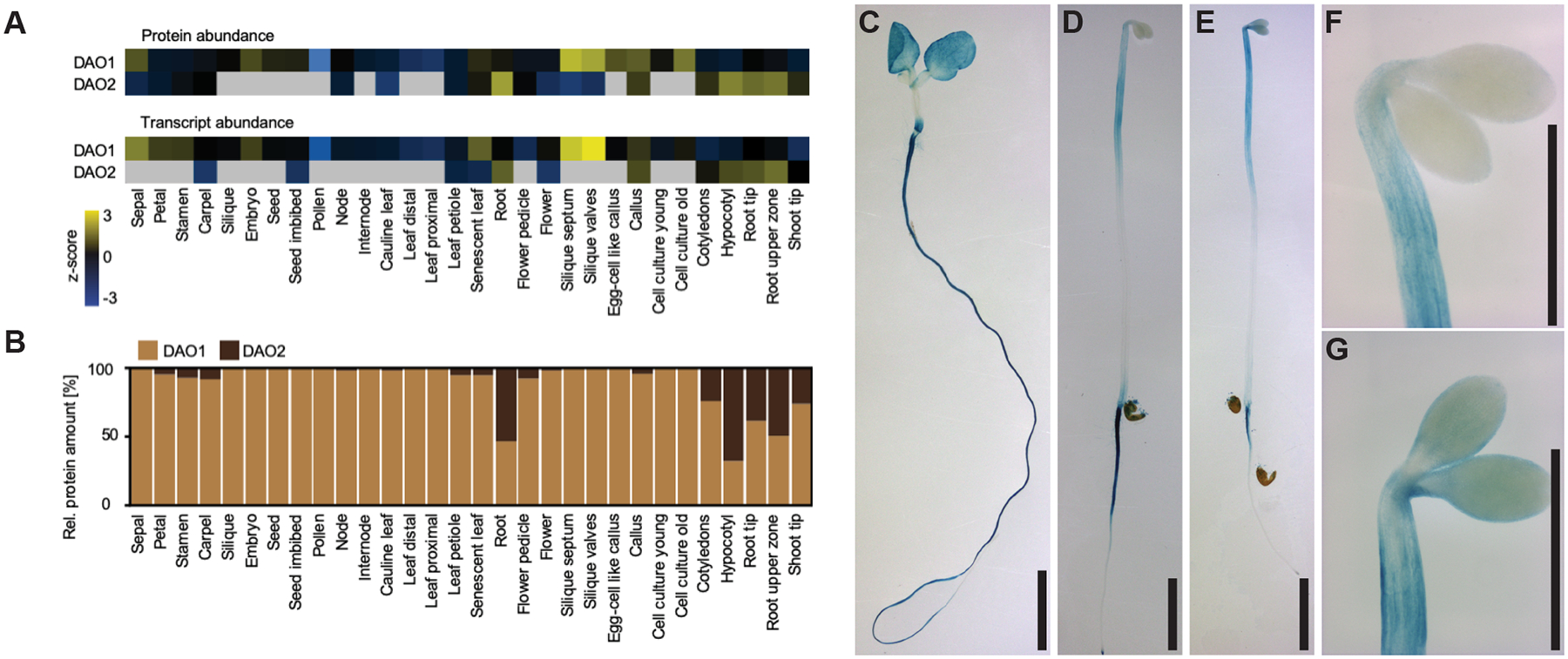
Spatio-temporal expression shows that DAO1 is ubiquitous and DAO2 is in roots and hypocotyls. **(A)** Heatmap of z-scored protein and transcript expression profiles for *DAO1* and *DAO2* across 30 tissues. (**B**) Comparison of relative protein amounts of DAO1 and DAO2 within each tissue. **(C-F)** GUS staining in *DAO1*:GUS and *DAO2*:GUS seedlings. (**C)** 7-day light grown *DAO2*:GUS. (**D)** 3-day etiolated *DAO1*:GUS. (**E)** 3-day etiolated *DAO2*:GUS. **F** Apical region of 3-day etiolated *DAO1*:GUS. (**G)** Apical region of 3-day etiolated *DAO2*:GUS. Scale bars: 1 mm.

*DAO1* and *DAO2* tissue-specific mRNA expression patterns and protein profiles were examined across 30 tissues of plants grown under continuous white light (Mergner et al, 2020) (Fig. 3A). *DAO1* transcripts were ubiquitous, whereas *DAO2* was expressed in specific tissues, particularly the hypocotyl, root, and root tip of 7 d seedlings (Fig. 3A) and germinating seeds, consistent with microarray and RNAseq expression data (Winter et al., 2007; Kleipikova et al., 2016) (Table S5). No miRNAs were reported in the miR database for either *DAO1* or *DAO2* (miRBase.org). The protein abundance corresponded with the transcript abundance (Fig. 3A,B), consistent with previous findings of stronger *DAO1*:YFP-DAO1 fluorescence signal compared to weak *DAO2*:YFP-DAO2 signal (Zhang et al., 2016).

In 7 d seedlings grown in continuous light, *DAO1*:GUS activity was observed in cotyledons, hypocotyl, and primary root (Zhang et al., 2026; Porco et al., 2016), and *DAO2*:GUS signals were similar, but more discrete and less ubiquitous (Fig. 3C). In 3 d etiolated *DAO*:GUS and *DAO2*:GUS seedlings, GUS staining patterns were similar in upper hypocotyls, root-shoot transition zone and the primary root (Fig. 3D,E) with stronger *DAO2*:GUS activity in cotyledons compared to *DAO1*:GUS (Fig. 3F,G). *DAO2*:GUS activity appeared to be particularly strong on the concave side of the apical hook, similar to YFP-DAO1 (Zhang et al., 2016).

#### Additive and unique phenotypes of dao1 and dao2

Germination rates of fresh seeds in *dao* single and double mutants were examined, and no differences were observed among wild type, *dao1*, and *dao1 dao2* seeds (Table 1). However, germination of fresh *dao2* seeds was significantly delayed compared to the other genotypes at 4 days after sowing (P < 0.01, Table 2), but the *dao2* seeds germinated later; the germination delay was ameliorated with after-ripening.

**Figure 4.**
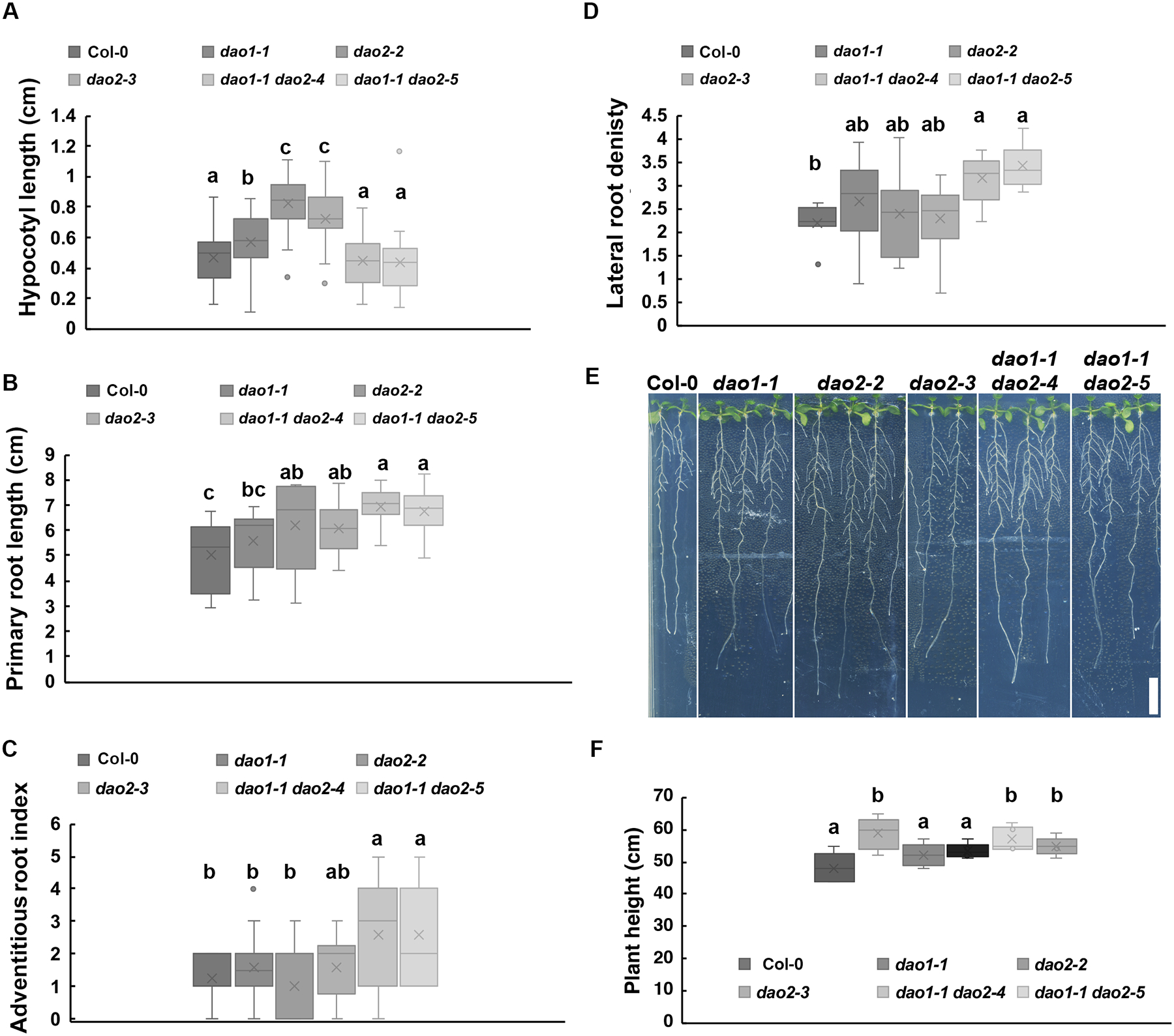
Phenotypes of *dao* alleles support synergistic function in lateral and adventitious root numbers. Col-0, *dao1-1*, *dao2-2*, *dao2-3*, *dao1-1 dao2-4* and *dao1-1 dao2-5* seedlings were analyzed. (**A)** Hypocotyl length in 2-day-old etiolated seedlings. (**B)** Primary root length, (**C)** adventitious root index, and **(D)** lateral root density of 10-day old light-grown seedlings. **(E)** Images of 10-day old seedlings. **(F)**. Inflorescence height of 43-day-old plants. Bars indicate SD. Scale bar, 30 cm. Data shown are means ± SE (n ≥ 10). Letters indicate statistical differences by ANOVA followed by Tukey’s post-hoc analysis (P < 0.05). Scale bar: 1 cm.

In 2 d etiolated seedlings, *dao1* and *dao2* single mutants had significantly longer hypocotyls compared to wild type and double mutants (P < 0.01; Fig. 4A), and these differences decreased as the seedlings matured, as observed for *dao1* in Zhang et al. (2016) (Fig. S5). In 10 d light-grown seedlings, primary root lengths in *dao2* and *dao1 dao2* were significantly longer than wild type (P < 0.05; Fig. 4B). The *dao1* root lengths were variably longer but not statistically different from wild type or *dao2* under these growth conditions (Fig. 4B). Lateral root density and the adventitious root index were significantly greater in the *dao1 dao2* double mutants than wild type (P < 0.05) similar to Lakehal et al. (2019), but single mutants were not different from wild type under the conditions used here (Fig. 4C–E), in contrast to the increase observed in *dao1* adventitious root index in Lakehal et al. (2019). During vegetative growth under long-day growth conditions, adult *dao1* and *dao1 dao2* were taller than wild type and *dao2* lines (Fig. 4F; Fig. S6).

During reproductive growth, *dao1 dao2* double mutants showed wild type 4-petaled flowers (P > 0.5) and a slightly increased number of flowers with altered petal numbers (3 or 5 petals) compared to wild type (P < 0.05; Table 2; Fig. S7), perhaps due to changes in local auxin maxima in the petal primordia initiation region. Since auxin biosynthesis increases with an increase in temperature (Gray et al., 1998; Bianchimano et al., 2023), adult plants with inflorescences 10 cm high were shifted from 22 °C to 26 °C for 12 h (see Methods), and then the petals on each flower were counted 9.5 d later. The number of 4-petaled flowers was the same as wild type, but a significant increase in the number of 3- or 5-petaled flowers was observed (P < 0.05; Table 2). No carryover of heat stress was observed, and the flower numbers returned to those observed at 22 °C.

Overall, loss of both genes has a small but additive effect in the roots where the expression of the genes overlaps: the numbers lateral and adventitious roots and root length, but otherwise resembled either single mutant. Loss of both genes also had a temperature-dependent additive effect resulting in alteration of petal number compared to wild type. The observed phenotypic difference observed in transitional tissues where auxin transiently accumulates is consistent with *DAO* expression and homeostatic function.

### The etiolated apical hook as system to study DAO and oxIAA

#### IAA metabolism in the apical hook

Apical hook formation and opening in etiolated seedlings was chosen as a system to further assess the physiological role of DAO and oxIAA. IAA accumulations resulting from synthesis and transport have been shown to interact with cell wall modification processes to regulate apical hook formation (Žádníková et al., 2010; Walia and Carter et al., 2024). Auxin accumulation results in apoplastic acidification that, in turn, enhances localized cellular auxin accumulation until acidification directly inhibits cellular elongation. This phenomenon is readily observable in roots, but generally requires higher auxin accumulations in shoots under normal growth conditions due to differential cell wall/cuticle composition. A strong DR5::GUS signal is readily observed during formation of the apical hook of Col-0 and attenuates during a quiescent period before a default dark “last resort” response triggers hook opening (Žádníková et al., 2010).

Seedlings were grown synchronously, handled and imaged with rigorous control of temperature and light (see Methods). Although the DII-VENUS auxin reporter, which exhibits decreased signals in presence of IAA, is somewhat difficult to visualize in etiolated hypocotyls (Hohm et al., 2014; Sakai, 2019), it is consistently absent on the concave side of the apical hook during formation but reappears transiently at the onset of the hook maintenance period (Fig. S9). As DR5 reporters are dependent on transcriptional activity and natural turnover of the fluorescent protein tag (Hayashi et al., 2014), and DII-VENUS is a fluorescent protein fusion to the AUX-IAA co-receptor degron with signal loss reporting auxin-dependent proteolysis (Brunoud et al., 2012), the comparative latency of DR5 reporter over a few hours is expected.

**Figure 5.**
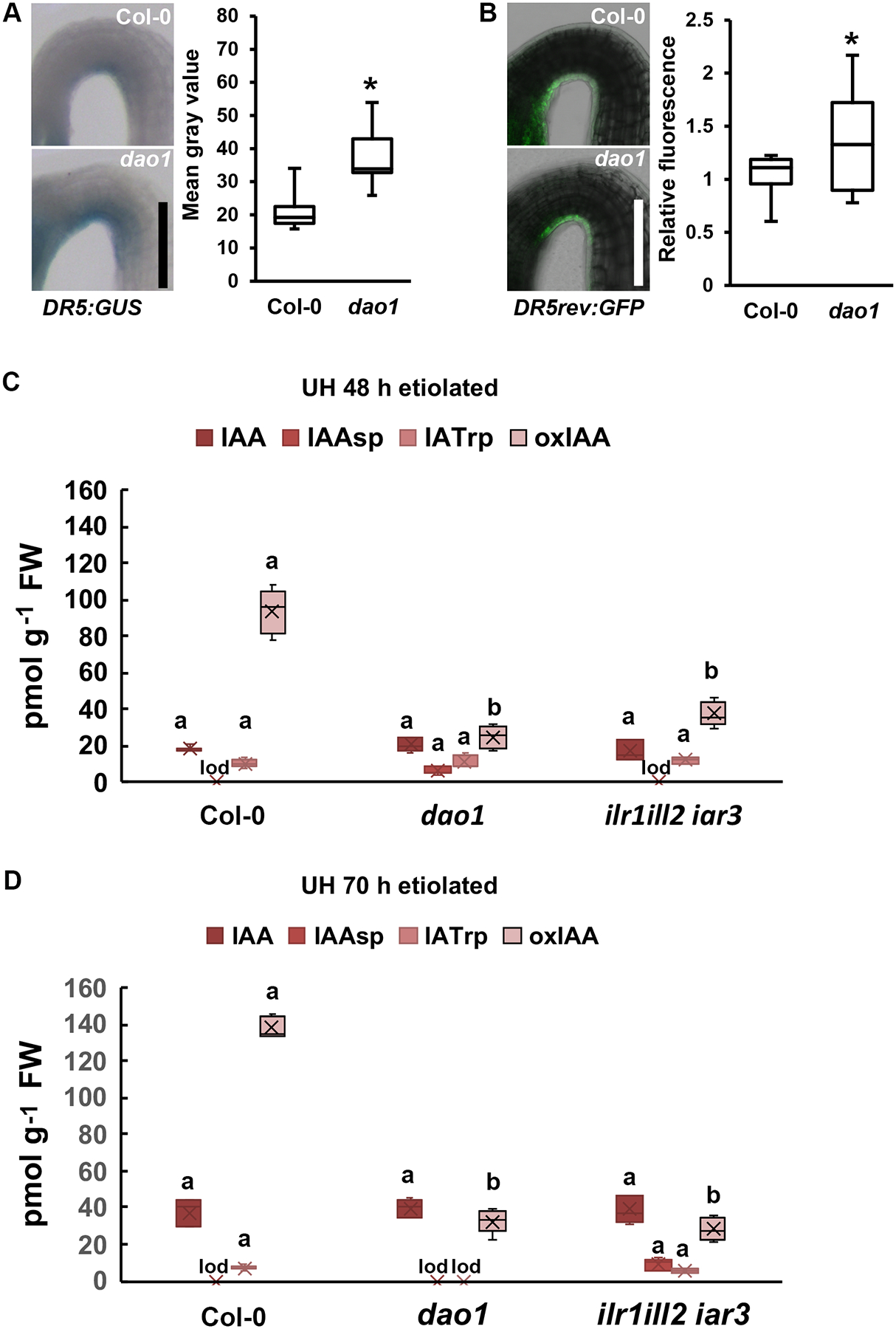
IAA levels in etiolated seedling UH/hooks are low at 48 h and increase by 70 h. **(A)** *DR5:GUS* in Col-0 and *dao1* apical hooks at 2 d. Quantification of *DR5:GUS* signals (right panel) (n=10). **(B)** *DR5rev:GFP* in Col-0 and *dao1* apical hooks at 2 d. Quantification of *DR5rev:GFP* (right panel) (n=12). (A-B) Asterisks indicate statistical difference by two-tailed Student’s *t*-test (P<0.05). Scale bars: 0.2 mm. (**C-D)** IAA, IAAsp, IATrp, and oxIAA in the upper hypocotyl (UH) of **(C)** 48 h and **(D)** 72 h etiolated hypocotyls. UH includes cotyledons, hook, and apical hypocotyl region at the point equivalent to where the cotyledon tips end. (n=5). Letters indicate statistical differences by ANOVA followed by Tuley’s post-hoc analysis (P<0.05) “lod”, limit of detection.

Previously, etiolated *dao1-1* mutants were shown to have differential apical hook angle and phototropism (Zhang et al., 2016), while the complemented *dao1-1* (Zhang et al., 2016), and *dao2*, showed wild type hook angles (Col-0 178 ± 1.96, complemented *dao1* 178 ± 1.17; Fig. S8). DAO1 and DAO2 signals were observed on the concave side of the apical hook (Fig. 3D–G), and stronger DR5:GUS and DR5rev:GFP signals within *dao1* hooks compared to wild type (P < 0.05; Fig. 5A,B). LC-MS quantitations of the apical hook-upper hypocotyl region (referred to as upper hypocotyls) in etiolated seedlings at 48 h and 70 h after hook emergence confirmed this transition in auxin metabolite status. By 48 h, IAA levels in all genotypes had decreased to very low levels. Between 48 h and 70 h, Col-0 IAA levels increased 2.7-fold, while oxIAA increased only 1.4-fold (Fig. 5C,D). At 48 h, auxin levels in *dao1* were marginally higher than wild type, and were 1.3-fold higher at 72 h. *ilr1 ill2 iar3* IAA levels were the same as wild type at 48 h, and the same as *dao1* at 72 h. IAAsp but not IAGlu, was consistently detected as the primary IAA conjugative metabolite in these assays, and levels of oxIAA and IAAsp were as expected in the loss-of-function lines (Fig. 5C,D; Hayashi et al., 2021). The detection of low levels of IATrp in the upper hypocotyls of all genotypes at the time of IAA minima coincided with higher Trp levels observed with reduced consumption in downstream primary and specialized metabolic pathways. However, IATrp has also been associated with inhibition of auxin signaling (Staswick et al., 2009).

A time-course of auxin metabolite levels measured in etiolated seedling upper and lower hypocotyls exposed to light for 6 h at the quiescence and transition to opening stages demonstrated low and then slowly increasing IAA levels with oxIAA levels much higher. With light exposure at 2 d (48 h), IAA levels in upper hypocotyls increased significantly and oxIAA levels remained constant over 6 hours (Fig. S10A). When exposed to light at 3 d (70 h), significant increases in IAA were observed, and oxIAA levels decreased slightly over 6 hours (Fig. S10B). For comparison, IAA levels presumed to be derived from polar rootward transport were low in lower hypocotyls, but did increase in 3 d seedlings after 4 h light treatment (Fig. S10C,D).

#### Genetic and pharmacological evaluation of apical hook opening

**Figure 6.**
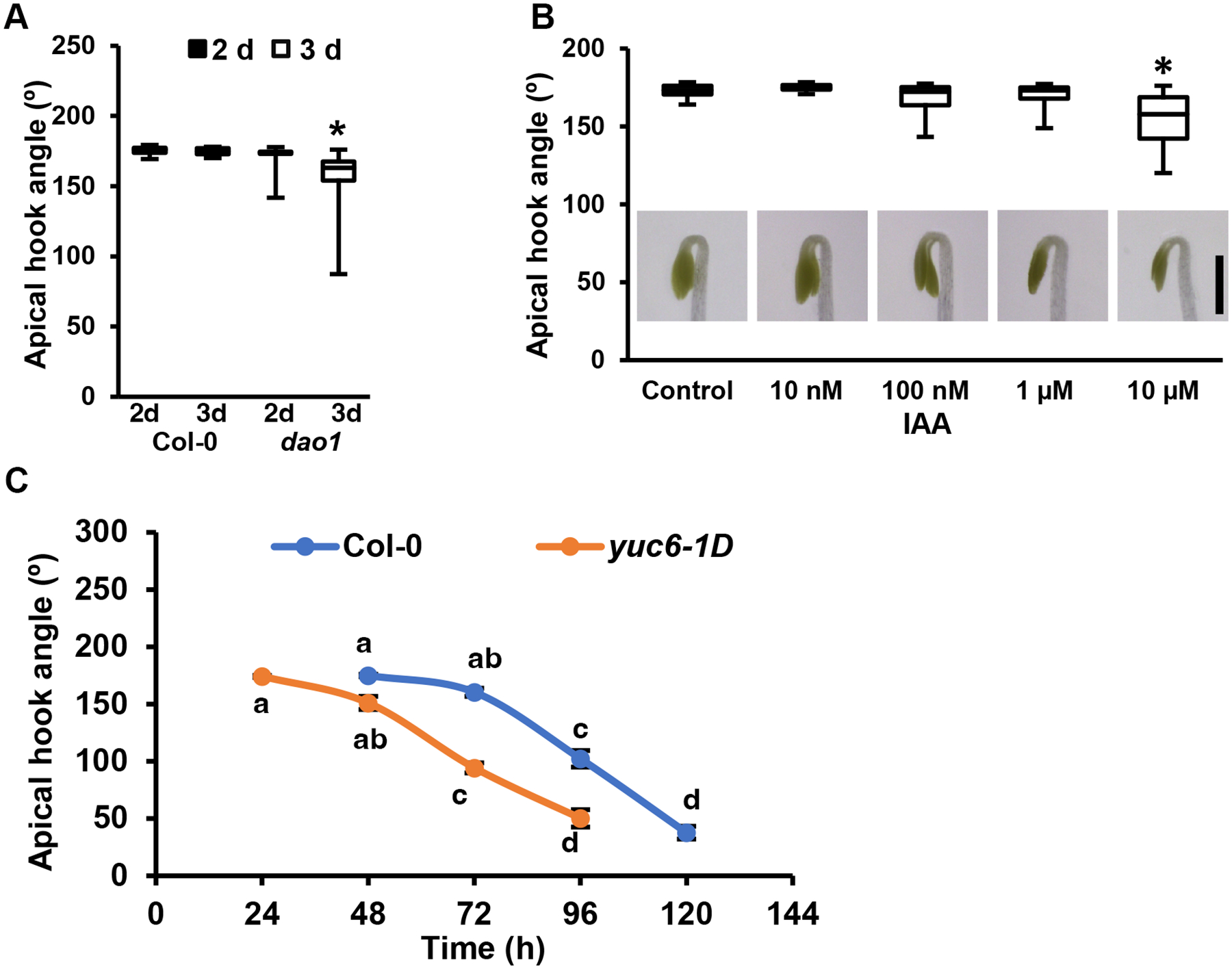
IAA increases apical hook angles in etiolated seedlings. **(A)** Apical hook angles of 2 d and 3 d Col-0 and *dao1* (n=15). Asterisks indicate statistical difference from Col-0 by Kruskal-Wallis (P<0.001) followed by Steels’s post-hoc analysis (P<0.05). **(B)** Apical hook angles of Col-0 treated with exogenous IAA (n=20). IAA was applied in lanolin paste at concentrations shown to the inner hook region of 48 h seedlings using a fine tip toothpick, then seedlings were grown an additional 24 h. Asterisks indicate statistical difference from control by Kruskal-Wallis (P<0.001) followed by Steels’s post-hoc analysis (P<0.05). Scale bar, 1 mm. **(C)** Kinetics of apical hook maintenance and opening in Col-0 and *yuc6-1D* mutants. Data shown are means ± SE (n=15). *yuc6-1D* hook angles were not statistically different from Col-0 measured at points 24 h earlier. Letters indicate statistical difference by ANOVA (P<0.001) followed by Tukey’s post-hoc analysis (P<0.05).

In 2 d and 3 d etiolated seedlings, the apical hooks in *dao1* were more open than Col-0 and were significantly different from wild type at 3 d (Fig. 6A), consistent with the observed increased in free IAA (Fig. 5C,D). IAA:oxIAA ratios were experimentally manipulated by exogenously applying these compounds. When auxin was applied in a small spot of lanolin to the inner hook of 2 d etiolated Col-0 seedlings, the degree of hook opening was dose-dependent, and 10 μM IAA phenocopied the null *dao* mutants (Fig. 6B). In Col-0, artificial pooling of auxin in the apical hook induced by application of the auxin transport inhibitor NPA to increase IAA and oxIAA over 24 h (Fig. S11A) also resulted in relaxation of apical hook angles that were nearly open after 12 h and were highly variable by 24 h (Fig. S11B).

The transitional nature of auxin-induced hook opening was assessed in etiolated auxin-overproducing *yuc6-1D* seedlings (Kim et al., 2007). Kinetic analyses showed that etiolated *yuc6-1D* hooks opened ~24 h earlier than in Col-0 (Fig. 6C), but were not different from Col-0 after an 8 h light treatment despite a greater initial hook angle (Fig. S12).

As oxIAA uptake has been shown in Arabidopsis seedling roots, presumably via AUX1/LAX proteins (Kubeš et al., 2012; Fig. S13), 5 μM oxIAA in lanolin was applied to the concave surface of apical hooks of 2 d etiolated seedlings. When etiolated Col-0 apical hooks were treated with IAA (Fig. 6B), hook opening was induced, while oxIAA had no effect (Fig. S14A). In *dao1* IAA treatment resulted in rapid opening (Fig. S15). oxIAA application at the time when IAA levels were at their minimum slightly delayed the onset of hook opening in the dark in both Col-0 and *dao1-1 dao2-4*, while treatment with a very high concentration (50 μM) of oxIAA was able to partially restore *dao1* to hook angles close to those of Col-0 (Fig. S15C).

### oxIAA and auxin signaling

The observed kinetics of IAA catabolism and oxIAA formation suggest that oxIAA may, indeed, act as a weak anti-auxin with effects only evident under conditions where cellular IAA levels are very low. IAA acts as a ‘molecular glue’ to enhance interaction of AUX/IAA transcriptional repressor proteins with TIR1 (Cao et al., 2022; Tan et al., 2007). This interaction results in SCF^TIR1^-mediated poly-ubiquitination and degradation of the AUX/IAA proteins, (Gray et al., 2001; Dharmasiri et al., 2005; Kepinski and Leyser, 2005; Shabek et al., 2014), and results in gene expression that include regulators of cell expansion (Fendrych et al., 2016). oxIAA has previously been shown to enhance the interaction between TIR1 and IAA7/AXR2 (Pěnčík et al., 2013), suggesting oxIAA may have a weak biological activity.

**Fig 7.**
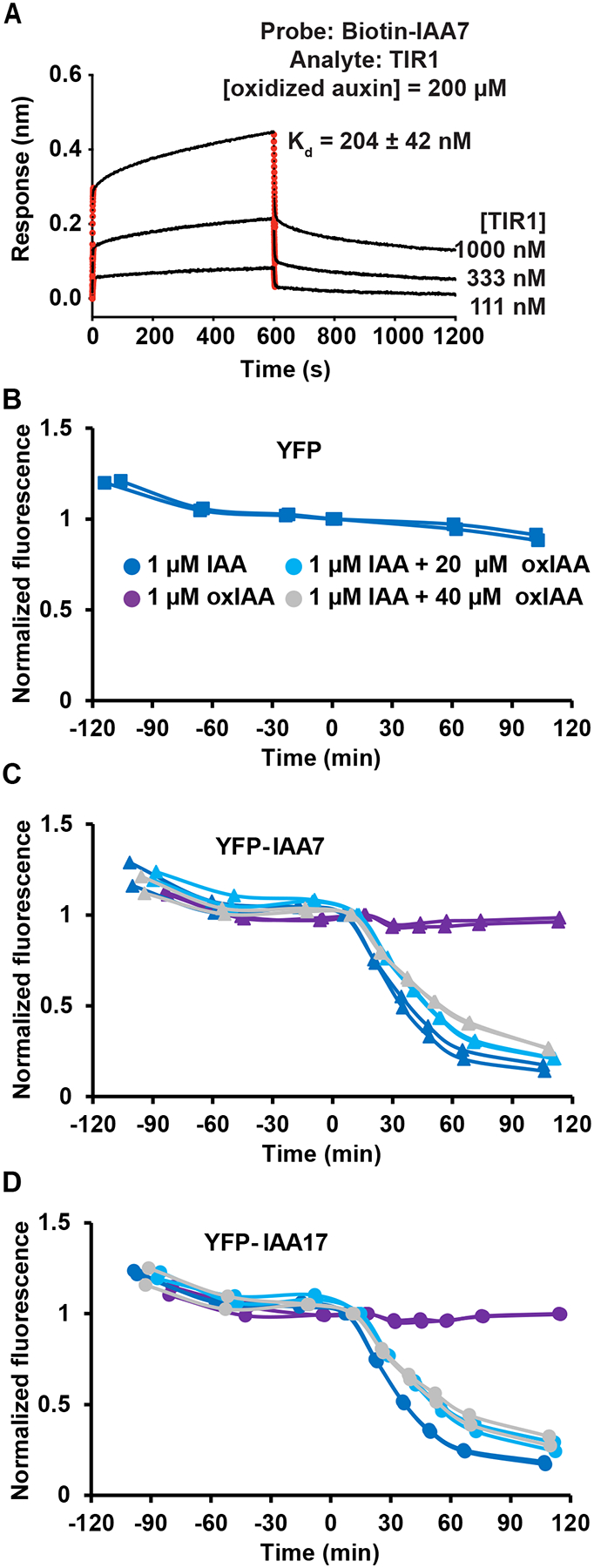
oxIAA can bind TIR1 and IAA7/IAA17 with ~10x less affinity than IAA. **(A)** BLI analysis of IAA7-TIR1 binding affinity of in the presence of oxIAA at a saturating concentration. Red lines represent curve fitting at the initial phase of association and dissociation. *K*_d_, dissociation constant. Assay was repeated twice with similar results. **(B-D)** Effects of oxIAA on IAA-dependent degradation of **(B)** YFP alone and **(C)** YFP-IAA7 and **(D)** YFP-IAA17. IAA and oxIAA were added at time 0. Data from two independent replicates are plotted together. The symbols are Squares: YFP alone; Triangles: YFP-IAA7; Circles YFP-IAA17.

To test this, IAA7-TIR1 binding assays were repeated using a BioLayer Interferometry (BLI) assay (Cao et al., 2022). Under saturating concentrations, oxIAA enhanced affinity between IAA7 and TIR1 from 20 μM (Cao et al., 2022) to 200 μM (Fig. 7A). This suggests that oxIAA acts as a molecular glue to enhance IAA7-TIR1 interaction, but with less activity than IAA, which enhanced IAA7-TIR1 affinity to ~30 nM (Cao et. al., 2022).

Canonical auxin-mediated transcriptional activation involves rapid TIR1/AFB generation of cAMP second messengers and slower AUX/IAA ubiquitination and subsequent degradation after formation of an AUX/IAA-TIR1 complex (Chen, et al., 2025). oxIAA was therefore examined for initiation of TIR1-dependent degradation of IAA7 and its paralog IAA17 using a synthetic yeast-based fluorescence system (Havens et al., 2012). While no signal degradation of YFP alone was observed (Fig. 7B), signals from YFP-IAA7 and YFP-IAA17 decreased rapidly upon addition of IAA (Fig. 7C,D). Addition of oxIAA had no effect alone, however, co-treatment of oxIAA with IAA slowed the rate of IAA7 and IAA17 degradation (Fig. 7C,D). This suggests that when IAA levels are low, oxIAA competes with IAA for TIR1 binding, but oxIAA-bound TIR1 does not result in IAA7 or IAA17 degradation in yeast.

Hook opening in IAA7 and IAA17 mutants was examined. The IAA7 dominant, gain-of-function mutant *axr2-1* has a point mutation that prevents IAA7 association with TIR1 (Timpte et al., 1994; Nagpal et al., 2000). The *axr2-1* mutant initiated hook opening prematurely (Fig. S15A), as observed in Pěnčík et al. (2013). The apical hooks of *iaa7* and *iaa17* loss-of-function mutants also opened early, but late-stage kinetics varied. While *axr2-1* hooks continued to open rapidly, *iaa7* and *iaa17* hook opening slowed between 72 and 96 h (Fig. S15A).

IAA treatment induced hook opening more rapidly in *dao1* (Fig. S15C). In *iaa7* IAA-induced hook opening was similar to Col-0, but oxIAA did rescue the phenotype (Fig. S15A,B,D). These results suggest that oxIAA weakly enhances IAA7-TIR1 interactions at the apical hook when IAA levels are extremely low. oxIAA may weakly bind other AFBs to enhance AUX/IAA interaction in cells with low IAA levels, but would be readily competed away under normal conditions. The slow rate of DR5 reporter activation after oxIAA treatment suggests no activation of TIR1/AFB cAMP formation, although an inhibitory impact cannot be ruled out.

## Discussion

We set out to resolve a number of outstanding questions posed by twentieth century publications and our own discoveries that explored oxidative inactivation of auxin: 1. Can the membrane-associated auxin oxidase activity reported in that literature be attributed to DAO? 2. Are DAO1 and DAO2 activities largely redundant? 3. Does oxIAA function as an anti-auxin? The experiments presented herein largely resolve those questions.

### 1. DAO1 is both cytosolic and membrane resident.

Membrane association of functional fluorescent fusions of DAO1 have been observed in Arabidopsis and tobacco BY2 cells using confocal laser scanning microscopy (Zhang et al., 2016; Müller et al., 2021). Western blot analysis shows a majority of DAO1 associated with microsomal fractions equivalent to those associated with monomeric status (Fig. 1), but becomes more soluble after auxin treatments similar to those that induce dimer formation (Takehara et al., 2021). The likelihood of preferential IAA complexation with membrane-resident DAO1 and experimental limits wherein <15% of IAA substrate was converted before ATP exhaustion at 30 min require cautious interpretation of these assays. The evidence presented here suggests that DAO1 residence at the membrane involves interactions with other proteins, presumably via two putative μ-adaptin domains and an actin binding cleft (Zhang et al., 2016). The modest increase in apparent IAA oxidation by membrane-resident DAO1 suggests a possible role for slow oxidation in moderating or attenuating plasma membrane localized transport or signaling events.

### 2. DAO2 is enzymatically similar to DAO1 and helps regulate auxin homeostasis when auxin levels are high.

Experimental evidence presented here demonstrates that DAO2 activity against IAAsp is similar to DAO1 (Table S3), and likely accounts for the residual 5–10% oxIAA formation observed in *dao1-1* roots (Fig. 2). Under the conditions used here, *dao1 dao2* null mutants also have more lateral organs (lateral roots, adventitious roots) than either mutant alone (Fig. 4). This minor contribution under normal growth conditions is underscored by the observation that GH3-mediated conjugation increases only when *DAO1* is non-functional (Fig. S1) (Zhang et al., 2016; Porco et al., 2016). However, changes in observed auxin metabolite levels observed in *dao2* single mutants suggest that DAO2 enzymatic activity may have tissue-specific relevance.

Both floral development and IAA/oxIAA biosynthesis are regulated by circadian processes (Fowler et al., 1999; Covington and Harmer, 2007; Voß et al., 2015). As such, increases in auxin production due to temperature could impact synchronization of new organ initiation. The initiation of floral organ primordia and specific phyllotaxy is dependent on precise meristematic auxin maxima, and local changes via exogenous auxin application can produce new floral organs (Reinhardt et al., 2000). Floral organ boundaries are also controlled by regulation of auxin biosynthesis, and *DR5* signals are seen at the petal primordia initiation sites (Xu et al., 2018). Low levels of auxin induce *CUP-SHAPED COTYLEDONS 2* (*CUC2*), which defines the petal primordia boundary in floral meristems, but high levels of auxin repress its expression (Heisler et al., 2005). In the double *dao1 dao2* mutants, decreased homeostatic regulation of auxin levels perturbed by increased temperature appears to interfere with the precision of normal boundary and petal primordia initiation, likely in stage 3/4 flowers, with the result of altered petal number (Table 2).

### 3. oxIAA does function as a weak anti-auxin

The *in vitro* and *in vivo* results shown herein are consistent with oxIAA exhibiting weak anti-auxin activity under quiescent low auxin conditions. In the system evaluated herein, IAA was readily detectable and presumably concentrated in the concave region of the apical hook as has been described previously (Žádníková et al., 2010) during apical hook formation and initial skotomorphogenic hypocotyl elongation. Subsequently, IAA synthesis decreases and reciprocal conjugation and oxidation increase (Fig. 5, Fig. S10) as the seedling is poised to receive the first photons to induce auxin-mediated hook opening and cotyledon expansion. During these processes, DAO1 signals are enhanced on the concave side of the hook (Zhang et al., 2016), and oxIAA levels remain several fold higher than IAA (Fig. 5, S10). This suggests that auxin oxidation via DAO1 may contribute to the temporary suspension of auxin signaling during the pause before hook opening. The competitive advantage of IAA in stimulating auxin action is demonstrated when localized IAA application to the concave side of the apical hook induces opening (Fig. 6) or when IAA levels rapidly increase with light treatment (Fig. S10). Although “last resort” programming that initiates apical hook opening and cotyledon expansion to potentially absorb light is triggered by an uptick in auxin production after ~24 h without further stimulus, this mechanism is of limited significance as Arabidopsis seedlings rapidly fail without light unless grown on hexose-supplemented media.

Previously, the artificial auxin RN4 used as an “anti-auxin” agonist to investigate apical hook opening. Vain et al., showed that IAA17 was needed for hook formation and IAA7 needed to be degraded during the maintenance phase; further, RN4 enhanced IAA3- and IAA7-TIR1 interactions but decreased IAA1- and IAA17-TIR1 interactions. This suggests IAA and analogs can bind TIR1 to alter individual AUX/IAA interactions with TIR1 (Vain et al., 2019). Here both IAA7 and IAA17 were stabilized in the presence of oxIAA and the interaction with TIR1 is maintained (Fig. 7). Although clearly a weak auxin/anti-auxin, oxIAA has been shown to stimulate cell expansion at high concentrations (Galston and Chen, 1965) and to enhance interaction between TIR1 and IAA7 (Pěnčík et al., 2013). Some accounts of oxIAA activity could be a result of IAA contamination as was found in a reagent used in preliminary experiments for this study. Although application of 25 nM IAA had no impact on hook opening, the reagent was replaced with another lot with IAA contamination at or below the limit of detection. Specific biophysical measurement by Bio-Layer Interferometry revealed oxIAA does act as a ‘molecular glue’ to enhance TIR1-IAA7 interaction (Fig. 7), but with considerably less affinity than with IAA (Cao et al., 2022). While oxIAA alone did not initiate TIR1-dependent IAA7/17 degradation in yeast, oxIAA did slow the rate of IAA-induced degradation (Fig. 7).

These data suggest that oxIAA acts as a weak TIR1-binding auxin that de-represses a subset of AUX/IAA regulated genes but without (or at least diminished) AUX/IAA degradation. This low-level activity is consistent with oxIAA contributing to a delay in apical hook opening (albeit at very high concentrations), and only before onset of light- or time-dependent increases in intracellular IAA levels that easily displace and out-compete oxIAA. Interactions of oxIAA with AFB1, a member of the TIR1/AFB gene family that was recently demonstrated to negatively regulate auxin-induced gene expression via cytoplasmic interactions (Dubey et al., 2023) should be explored. Further, low affinity inhibition of TIR/AFB adenylate cyclase activity by oxIAA should be explored.

Considering the physiological responses associated with DAO activity as well as its partial membrane residence, a potential target oxIAA would be the ABP1/ABL-TMK membrane-localized receptors. Modelling of the ABP1/ABL auxin binding pocket with AlphaFold-2 (Murphy and Jones, 2023) does suggest that oxIAA would fit loosely, but not be excluded at extracellular pH. However, there is no evidence that oxIAA is found in the apoplast, and previous efforts to identify plasma membrane export were unsuccessful (Kubeš et al., 2012). As such, interaction of oxIAA with the cell surface receptor system is unlikely. In our hands, *tmk1* and, to a lesser extent, *tmk4* (but not *abp1*) mutants exhibit the reduced apical hook quiescence that has been described (Fig. S14; Cao et al., 2019). Application of oxIAA at 40 h in the timetable used here, slightly enhanced hook curvature of *tmk1* and resulted in less delay in hook opening compared to wild type (Fig. S14C). Although this result suggests that oxIAA targets are primarily nuclear, interactions with intracellular and cell surface ABP1/ABL targets require further study. It’s also possible that in the cytosol, oxIAA can compete with IAA for GH3 binding (Brunoni et al., 2023).

The results herein lead us to conclude that DAO isoforms are, in fact, responsible for most, if not all of the physiologically-relevant auxin oxidation described in classic papers that identified the activity. It is also likely that oxIAA functions as a very weak auxin that can repress auxin signaling during quiescent intervals, but be readily displaced by even modest increases in cellular IAA levels. While oxIAA should not be recognized as a primary signaling molecule, its observed modest anti-auxin activity stands as a reminder of the analog and slightly messy nature of the biochemical reactions that underly highly evolved cellular signaling mechanisms as well as the importance of this imprecision to evolutionary processes.

## Materials and Methods

### Plant material and growth conditions.

All lines (Tables S6, S7) were in the Columbia-0 (Col-0) background. Surface sterilized seeds were sown on 1/4 Murashige and Skoog medium (pH 5.6; Caisson Labs, Smithfield, UT, USA) containing 1 g L^−1^ 2-(N-morpholino) ethanesulfonic acid (MES), 0.5% sucrose, and 0.8% agar (for Fig. 3 and 4) or 1% phytagel and stratified at 4 °C for 3 d. For seedlings assays: Seedlings were grown under continuous 100 μmol m^−2^ s^−1^ light at 22 °C, except as indicated for specific treatments. Plants on soil were sown in a pre-watered soil mix (Sun Gro Propagation Mix, Sun Gro Horticulture, Agawam, MA, USA.), were stratified at 4°C 2 d and grown in growth chambers at 22 °C at 100 μmol m^−2^ s^−1^ light (16 h light/8 h darkness) unless otherwise indicated. ***For etiolated seedling experiments***: Unless otherwise specified, seeds were sown as above and stratified in the dark for 3 days; plates were placed under 90 μmol m^−2^ s^−1^ light at 22 °C for 6–8 h to induce germination then grown vertically in the dark for the times indicated. For light induction, seedlings were placed under 100 μmol m^−2^ s^−1^ white light at 22°C. Seedlings were monitored with a USB3 uEYE CP camera (IDS Imaging Development Systems, Woburn, MA, USA) under infrared light (Roitner LaseTechnik, Wien, Austria). Genotypes that exhibit initial agravitropic growth were placed in an upright position at 48 h. All incubations and applications described below were performed under dim green safe light.

### Microsomal membrane isolation of YFP-DAO1.

Col-0 and *DAO1*:YFP-DAO1 were grown on were grown on vertical plates as above for 5.5 d. For auxin treatment assays, seedlings were transferred to Whatman 3MM chromatography paper saturated with ¼ MS containing solvent control (ethanol), 100 nM IAA, or 1 μM IAA for 2 h just prior to microsomal membrane preparation. Soluble and microsomal proteins were isolated according to Yang and Murphy (2013) with modification of the grinding buffer. Seedlings were homogenized with mortar and pestle in grinding buffer consisting of 25 mM HEPES (pH 7.5), 5 mM EDTA, 0.29 M sucrose, 0.5% PVP 40,000, 0.2% BSA, 3 mM DTT, 100 μM PMSF, 200 μm benzamide/benzamidine, 0.2 μg/ml leupeptin, 2 μg/ml pepstatin A, and 0.1 μg ml^−1^ aprotinin. Soluble and membrane protein concentrations were determined using amido black. 15 μg total protein was incubated at 42 °C in 1X SDS-loading buffer for 20 mins prior to loading on a 12% SDS-PAGE gel. ***For western blot analysis:*** Proteins were transferred to nitrocellulose by wet transfer then incubated with a 1:5,000 dilution of α-GFP (Thermo Fisher Scientific, CAB4211) followed by 1:10,000 α-rabbit conjugated with horseradish peroxidase (Thermo Fisher Scientific, A27036). Proteins were detected using Bio Rad Clarity Max ECL substrate (Cat. #1705062S) and a BioRad ChemiDoc imaging system. Soluble and membrane percentages were determined by densitometry using ImageJ.

### Sucrose density fractionation.

YFP-DAO1 (Zhang et al., 2016), PIP2A-GFP (Cutler et al., 2000), YFP-RabA2A (Chow et al., 2008), VHA-A3-GFP (Dettmer et al., 2006), YFP-SEC12 (Agee et al., 2010) were grown on vertical plates for 5.5 d as above. Microsomal membrane isolation and sucrose density gradient was performed according to Yang and Murphy (2013) with the buffer modifications described above. 375 μl of 20, 25, 30, 35, 40, 45, 50, and 55% sucrose was layered in 13 × 51 mm ultracentrifuge tubes (Beckman Coulter, 349622) and incubated 4°C overnight to form a continuous gradient. Microsomal membranes (corresponding to 130 μg total protein) were layered on the gradient and centrifuged 100,000 × g, 4 °C, for 12 h. 375 μl fractions were collected and 25 μl used for SDS-PAGE. Western blot analyses were performed as described above.

### In vitro MYR of DAO1 N-terminal sequence.

Myristylation (Myr) assays followed by a MALDI–ToF analysis were performed as previously described (Castrec et al., 2018; Majeran et al., 2018; Monassa et al., 2023). Briefly, 100 μM of synthetic DAO1 peptide (GELNGVII) and positive control (GKQNSKLR) (Genscript, Piscataway, NJ) and 0.5 μM Arabidopsis NMT1 were incubated at 37°C in a reaction buffer [300 μL of a mixture containing 50 mM Tris (pH 8), 0. 193 mM EGTA, 1 mM MgCl_2_, 1 mM DTT, 5 μM sodium cholate, 0.04 mM Myr-CoA solution (stock solution 0.2 mM Myr-CoA, 10 mM sodium acetate, 2.5 μM sodium cholate)]. During the MYR reaction, 10 μL samples were collected a time 0 h and at 2 h, and then diluted in 90 μL of water/acetonitrile solution. Each sample was then diluted five times in the matrix solution [5 mg mL^−1^ of α-cyano-4-hydroxycinnamic acid solubilized in water/formic acid/acetonitrile (50/50/0.1%)]. 1 μL of each dilution was spotted on a metal target. Crystals were obtained using the dried droplet method and samples were analyzed using a MALDI-TOF-TOF 5800 (ABSciex) in positive ion mode. Survey scans were performed using delayed extraction (400 ns) in reflector mode for a total of 5 000 shots. Mass spectra were analyzed with Peakview software (ABSciex).

### CRISPR-Cas9 constructs transformation, screening and genotyping.

Guide RNA targeting *DAO2* was designed using the tools in the University of Arizona CRISPR-PLANT portal (genome.arizona.edu/crispr/CRISPRsearch.html). The guide RNA sequence was synthesized into two complimentary oligos. The oligos were then annealed and cloned into the CRISPR construct pHEE401 (addgene.org) following the protocol posted on the website. The construct was sequenced and then transformed into Agrobacterium strain GV3101. The gRNA constructs were transformed into Col-0 or *dao1-1* by *Agrobacterium tumefasciens* -mediated floral dip (Clough and Bent, 1998) to obtain single *dao2* and double *dao1 dao2* alleles. Two unique sequences, TCATCTCATATGGACGTTCA (+ strand) and ACCGATGTGTTACTAGGGAA (- strand) were used as guide RNAs to target *DAO2* in the *dao1-1* and Col-0 backgrounds, respectively. The resulting four independent null *dao2* alleles are: *dao2-2* and *dao2-3* in Col-0 containing a 243-nucleotide deletion and a single nucleotide deletion, respectively; and null *dao1-1 dao2-4* and *dao1-1 dao2-5*, containing a single nucleotide insertion and deletion, respectively. All gene edited lines contained frameshift mutations and were backcrossed to remove Cas9. While *DAO1* has one gene model, *DAO2* has three gene models that appear to have differences in the 5’ and 3’ untranslated regions (araport.org; Cheng et al., 2017) (Fig. S1D). The CRISPR-Cas9 lesions are downstream of the *DAO2* translational start, thereby affecting all three gene models.

### Quantitative Real Time-PCR.

Total RNA was extracted from 7 d seedlings using Trizol (Invitrogen) with lithium chloride precipitation. Complementary DNA (cDNA) was synthesized from total RNA (1 μg) using the SuperScript^™^ III First-Strand Synthesis System (Invitrogen). Quantitative Real-Time PCR was carried out usingSYBR Green PCR Master Mix (Applied Biosystem) and 10-fold diluted cDNA templates (synthesized above) on a Bio-Rad CFX96 Touch Real-Time PCR Detection System. Melting curves of SYBR green wells were cross checked to eliminate nonspecific amplification. Data are normalized to UBQ5 mRNA expression (internal control), and fold changes are displayed relative to control plant lines. Three biological replicates and three technical replicates were performed for each plant line.

### Enzyme Assays.

Enzyme assays with recombinant DO1 and DAO2 were performed as in Hayashi et al., (2021) with recombinant DAO1 and DAO2 prepared as previously described (Zhang et al., 2016). Activity assays of soluble and membrane fractions from Arabidopsis seedlings (prepared as described above) were conducted with 100 nmol IAA (Sigma, St. Louis, MO) or IAAsp (Olchemin, Olomouc, CZ) and 10 mg total protein in membrane and soluble protein preparations. Assay buffer was made fresh and added to fractions for final concentrations of 2mM BTP-MES (pH 7.5), 3 mM ascorbate, 5 mM 2-oxoglutarate, 0.5mM dithiothreitol, 0.5 mM NH_4_Fe(SO_4_)_2,_ and 2 mM MgSO_4._ ATP was added to a final concentration of 2.5 mM with labeled or cold IAA as indicated at the start of the assay. Assays were limited to the times indicated, as ATP signals detected Molecular Probes Assay kit (Thermo Fisher) returned to background levels at 20 min.

### Metabolite analyses.

Metabolite analyses for seedlings and enzyme assays with recombinant proteins were as previously described (Zhang et al., 2016) except that they were conducted on an Agilent 6470 Triple Quadrupole LC-MS at the EXPOSOME Facility University of Maryland in addition to the facility at the Ohio State University OARDC Metabolite Analysis Center. Standards, retention times, and mass transitions are listed in Table S2. Standard curves were parametric with unforced origins. Pilot experiments to determine conditions where any oxidative conversion of IAA was observed were conducted using ^3^H-IAA (American Radiolabeled Chemicals, St. Louis, MO, 50 pmol at 0.71 TBeq mmol^−1^ mixed with 100nmol cold IAA per 50μL reaction) and analysis on C-18 TLC plates using butanone:ethyl acetate:ethanol:water 3:5:1:1 as the mobile phase. Indolic (but not oxindolic) compounds and carboxylic acid compound densities were first visualized using Ehmann’s reagent (1:3 Ehrlich:Salkowski reagent) and bromocresol green. Rcf values of genuine standards were determine by visualization under UV light. Spots from assays were scraped from plates and measured in a scintillation counter.

### LC-MS/MS quantitation.

Analyses of auxin and auxin metabolites (oxIAA, IAAsp, etc.) were conducted as described previously (Zhang et al., 2016; Stasko et al., 2020) with the following exceptions. Mobile phase gradients were as previous, but used 2% methanol and 1% acetic acid in water for Solvent A. In low-mass tissue samples where aggregate levels of auxin were closer to the limits of detection and quantification, pipette tips were packed with Oasis solid phase extraction media (Waters, Milford MA, USA) and used at ¼ volume as described (Novak et al, 2012), and injection volumes were increased to 2 μL with sample run set to ΔEMV of 200 (+). A signal: noise ratio < 5 was set for the limit of detection (LOD) unless noted. In low-mass samples where IAA and IATrp levels were close to LOD/LOQ, additional runs focusing solely on the mass transitions for IAA and IATrp (and their respective standards) were run with increased dwell time to optimize resolution of these peaks. For low mass of samples, target metabolite peaks were quantified using ratiometric comparison to the peak areas of deuterated internal standards of known concentrations. Detectable levels of the IAA decarboxylation product indole-3-carbinol were identified in samples that included roots, but not in extracts from shoot tissues. LC-MS at OSU used Kinetex C-18 Phenomenex columns for separations. At Maryland, Poroshell (Agilent) HIC columns were used for separations.

LC-MS determinations of Col-0 were replicated using acetonitrile gradients as described by Hayashi et al., (2021) and were found to have similar results as reported. LC-MS Analysis of IAAsp standards found IAA contamination to be at or below LOD. A table of compound mass transitions, retention times, etc., are listed in Table S2.

### Tissue expression analysis.

Tissue expression data for *DAO1* (AT1G14130) and *DAO2* (AT1G14120) was extracted from a proteome and transcriptome analysis of 30 tissues from *Arabidopsis thaliana* (Ecotype Col-0) (Mergner et al., 2020). *DAO1* and *DAO2* abundances displayed as heatmaps were log2 transformed and z-scored for proteome and transcriptome, respectively. Relative protein amounts of DAO1 and DAO2 within each tissue sample are displayed as proportion of DAO1 and DAO2 of their combined iBAQ protein intensity.

### Histochemical GUS staining.

GUS staining was as described in Zhang et al. (2016) with modifications. Briefly, seedlings were incubated in GUS substrate for 1 hour at 37 °C and then destained in 70% ethanol followed by 90% ethanol prior to imaging for Fig. 3. For Fig. 6, tissues were incubated in 90% acetone at 4°C for 20 min then incubated in staining solution [50 mM sodium phosphate buffer (pH 7.0), 0.1% Triton X-100, 0.5 mM potassium ferrocyanide, 0.5 mM potassium ferricyanide, and 1 mM X-gluc] in the dark at 37°C for 5 h. GUS solution was removed and replaced with 70% ethanol and kept at 4°C overnight before imaging. Images were collected on a Zeiss Stemi 2000-C with Luminera Infinity2 camera and software. Assays were repeated twice with similar results.

### Germination assay.

Seeds that were harvested on the same day were imbibed on water on a filter paper in a small petri dish. The petri dishes were placed at 4 °C, in the dark for two days. On the third day, the seeds were moved into the light, and were scored for germination 24 h later. Germination was defined as radicle emergence from the seed coat.

### Morphometric data.

Root length, lateral root density, hypocotyl length, and apical hook angle were as previously described (Zhang et al., 2016). *Adventitious root index*. Adventitious root assays were performed according to Gutierrez et al. (2009), with minor modifications. Briefly, surface sterilized seeds were sown and stratified in the dark for 2 d as above. Plates were moved to light for 12 h to induce germination then placed vertically in the dark for 48 h to allow for hypocotyl elongation. Plates were then transferred to 90 μmol m^−2^ s^−1^ continuous white light to induce adventitious rooting.

### Flowering time analysis.

In order to avoid transplanting-induced stresses that can alter flowering time, our analyses were performed according to the method reported by Sandhu et al., (2012) with a few modifications. All Arabidopsis seeds were directly sown in pots containing a pre-watered soil mix and stratified as above. The pots were transferred to a plant growth chamber (Conviron Model BDR8, Controlled Environment Ltd, Manitoba, Canada) set at 21 °C, 16 h light: 8 h darkness, 50% relative humidity and 100 μmol m^−2^ s^−1^ white light. At least 25 plants of each genotype grown in a randomized complete block design were used for flowering time analysis. Statistical analysis was performed using uncorrected Fisher’s LSD, p-value 0.05. Error bars are standard error of mean (SEM).

#### Petal number measurements

Arabidopsis seeds were directly sown in 2-inch pots as above with one plant per pot and one genotype per tray, 50 plants per tray, and seeds were stratified as above. Trays were transferred to 22 °C, 14-hour days and grown until inflorescences were 10 cm. Four trays of each genotype were shifted to 26 °C for 12 hours, and four trays remained in chamber at 22°C. Flowers were scored for 4 petals or for 3 or 5 petals (combined), 9.5 d following the temperature shift for both temperature treatments. Flowers lose petals over time, and petals lost to abscission and silique formation were not counted. Approximately, 1500 flowers with three replicates of each genotype were scored for petal number.

### Apical hook and hypocotyl measurements in etiolated seedlings.

For hook measurements, seedlings were removed from the dark at times indicated, carefully reoriented to ensure measurement accuracy, and then quickly imaged on a Zeiss Stemi 2000-C with Luminera Infinity2 camera and software. Hook angles between the hypocotyl axis and cotyledons were measured using ImageJ (Schneider et al. 2012). Assays were repeated three times with similar results.

### Hormone treatments.

Surface sterilized seeds were grown as above. For IAA and oxIAA treatments, 10 nM – 10 μM IAA (in ethanol) or solvent control was mixed into lanolin paste and a light layer was applied to the inner hook region using a fine tip toothpick. Hook angles were measured 24 h after application. Assays were repeated three times with similar results.

### Imaging by confocal laser scanning microscopy.

All confocal microscopy was performed using a Zeiss LSM 710 (Carl Zeiss, Jena, Germany) and fluorescence measurements made using Zeiss Zen software. DR5:GFP signals were visualized using a using a 20x objective with an excitation of 488 nm (5% laser strength) and emission of 495–588 nm. Fluorescence intensities were measured along 10 μm thick line traced on the concave side of the hook from the cotyledonary node down the hypocotyl. Assays were repeated twice with similar results.

### Bio-layer interferometry.

Bio-layer interferometry (BLI) was performed as previously described (Cao et al., 2022).

### Yeast YFP-IAA degradation assays.

Standard yeast drop-out and yeast extract-peptone-dextrose plus adenine (YPAD) media were used. Yeast strains (Table S8) were grown at 30 °C on appropriate selection plates (SDO-Tryptophan, -Leucine) for 2 d, and in SDO liquid media with 350 rpm in 10 mL culture tubes 16 hours to a concentration of approximately 200 events per microliter for cytometry analysis. Fluorescence measurements were taken using a Becton Dickinson (BD) special order cytometer with a 514 nm laser excitation fluorescence that is cut off at 525 nm prior to photomultiplier tube collection (BD, Franklin Lakes, NJ). Events were annotated, subset to singlet yeast using the FlowTime R package (github.com/wrightrc/flowTime). A total of at least 10,000 events above a 400,000 FSC-H threshold (to exclude debris) were collected for each sample and data exported as FCS 3.0 files for processing using the flowCore R software package and custom R scripts (PMC3440190, PMC5269309). Data from at least two independent replicates were combined and plotted in R (ggplot2.tidyverse.org).

### Statistical analysis.

Statistical analyses were conducted using Statistical Analysis Software (SAS) or Prism version 6 (Graphpad Software, San Diego, CA) for the phenotypes in *dao1* and *dao2* lines. For the etiolated seedlings, statistical analyses were performed as indicated in the figure legends using JMP PRO 17. Measurements were taken from individual samples. All data were tested for normality by Shapiro-Wilk test before applying statistical comparisons. Datapoints for fully closed and full opened Col-0 hooks are inherently skewed toward but not exceeding 180° and 0°, respectively, resulting in non-normal distributions. Therefore, non-parametric statistical comparisons for apical hook angles were made using Kruskal-Wallis followed by Steels’s post-hoc analysis.

## Supplementary Material

Supplementary Data Table S1. Fluorometric quantitation of DAO1-YFP in soluble and microsomal fractions with and without IAA treatment
Table S2. LC-MS/MS mass transitions and retention times of standards.
Table S3. Recombinant DAO1 and DAO2 activity.
Table S4. Native IAAsp vs IAA oxidase activity is predominant in soluble fractions and to a lesser extent in membrane fractions.
Table S5. Spatio-temporal transcriptome analyses of *DAO1* and *DAO2*.
Table S6. Accession numbers
Table S7. Lines used in this study
Table S8. Yeast strains used in this study
Figure S1. Construction of *dao2* CRISPR lines and expression in *dao1* mutants.
Figure S2. DAO2 is associated with soluble and membrane fractions.
Figure S3. Post-translation modification assays indicate N-terminal acetylation mediates DAO1 membrane residence.
Figure S4. IAA metabolites in roots and shoots of *dao* alleles grown in continuous light support primary oxidation of IAAsp.
Figure S5. Hypocotyl length in 4 d etiolated seedlings.
Figure S6. Plant height of *dao* mutants at 43 d.
Figure S7. Supernumerary petals in double mutants.
Figure S8. Complemented *dao1* hook and *dao2* hook.
Figure S9. DII-Venus signals in etiolated apical hooks are less persistent than DR5 signals.
Figure S10. IAA levels UH/hooks in etiolated seedlings increase with light.
Figure S11. NPA treatment increases IAA/oxIAA levels and relaxes apical hook angles at 12 h.
Figure S12. Auxin production in *yuc6-1D* alters developmental timing but not hypocotyl growth rate or light induced hook opening.
Figure S13. Protoplast loading of IAA and oxIAA at two different temperatures.
Figure S14. Exogenous oxIAA delays hook opening.
Figure S15. Apical hook opening in *dao1* is enhanced with IAA and retarded with oxIAA and hook opening in *iaa7* was restored to wild type with IAA but oxIAA treatment had little effect.

## Figures and Tables

**Table 1.** Germination frequencies among *dao* alleles. Seeds were scored for radical emergence four days after sowing. The remainder of the *dao2* seeds germinated later. Statistics for entire set: x^2^ (5, 1362) = 131.04, P < 0.005. The “-“ indicates that there is no reportable *x*^2^ or P value for Col-0 because Col-0 is the control.

Genotype	Total seeds sown	Germinated seeds	% Germination	*x* ^2^	P
Col-0	205	182	88.8	-	-
*dao1-1*	314	304	96.8	0.45	0.50
*dao2-2*	276	171	62.0	6.54	0.01
*dao2-3*	334	164	49.1	18.17	10^−4^
*dao1-1 dao2-4*	299	287	96.0	0.35	0.55
*dao1-1 dao2-5*	272	254	93.4	0.14	0.71

**Table 2.** Altered petal numbers are observed in *dao* alleles. Numbers of 4-petalled and 3/5-petalled flowers at 22 °C and 4-petalled and 3/5-petalled flowers 24 h after 12 h at 26 °C Data are means ± SD. Letters indicate statistical differences by ANOVA (P < 0.001) followed by Tukey’s post-hoc analysis (P < 0.05); *x*^*2*^ (4, 11801) = 23.15, *P* = 0.026

	22° C		26° C, 12 h	
	4 petal	3/5 petal	4 petal T shift	3/5 petal T shift
Col-0	1171.7 ± 5.0 ^a^	0.3 ± 0.3 ^a^	1168.7 ± 1.5^a^	1.0 ± 0.6 ^a^
*dao1-1*	1194.0 ± 4.7 ^b^	1.3 ± 0.7 ^a^	1175.0 ± 6.6 ^a^	4.3 ± 0.9 ^a^
*dao2-3*	1151.7 ± 15.9 ^a^	1.7 ± 0.6 ^a^	1147.3 ± 30.2 ^a^	7.3 ± 1.5 ^b^
*dao1-1 dao2-4*	1198.7 ± 3.8 ^b^	3.3 ± 0.7 ^b^	1176.7 ± 5.5 ^a^	17.0 ± 2.3 ^c^
*dao1-1 dao2-5*	1195.7 ± 5.0 ^b^	2.7 ± 0.3 ^b^	1167.0 ± 2.1 ^a^	15.7 ± 2.0 ^c^

## Data Availability

The data that support the findings of this study are available from the corresponding authors upon reasonable request.
