# Supplementary material for: Cytosolic- and membrane-localized oxidized indole-3-acetic acid formation regulates developmental auxin transients
Source: Plant Physiol. Author manuscript; Available in PMC 2025 Dec 23. (PMC12344411; doi:10.1093/plphys/kiaf330)
Supplement: Supplementary Data — Table S1. Fluorometric quantitation of DAO1-YFP in soluble and microsomal fractions with and without IAA treatment Table S2. LC-MS/MS mass transitions and retention times of standards. Table S3. Recombinant DAO1 and DAO2 activity. Table S4. Native IAAsp vs IAA oxidase activity is predominant in soluble fractions and to a lesser extent in membrane fractions. Table S5. Spatio-temporal transcriptome analyses of DAO1 and DAO2. Table S6. Accession numbers Table S7. Lines used in this study Table S8. Yeast strains used in this study Figure S1. Construction of dao2 CRISPR lines and expression in dao1 mutants. Figure S2. DAO2 is associated with soluble and membrane fractions. Figure S3. Post-translation modification assays indicate N-terminal acetylation mediates DAO1 membrane residence. Figure S4. IAA metabolites in roots and shoots of dao alleles grown in continuous light support primary oxidation of IAAsp. Figure S5. Hypocotyl length in 4 d etiolated seedlings. Figure S6. Plant height of dao mutants at 43 d. Figure S7. Supernumerary petals in double mutants. Figure S8. Complemented dao1 hook and dao2 hook. Figure S9. DII-Venus signals in etiolated apical hooks are less persistent than DR5 signals. Figure S10. IAA levels UH/hooks in etiolated seedlings increase with light. Figure S11. NPA treatment increases IAA/oxIAA levels and relaxes apical hook angles at 12 h. Figure S12. Auxin production in yuc6-1D alters developmental timing but not hypocotyl growth rate or light induced hook opening. Figure S13. Protoplast loading of IAA and oxIAA at two different temperatures. Figure S14. Exogenous oxIAA delays hook opening. Figure S15. Apical hook opening in dao1 is enhanced with IAA and retarded with oxIAA and hook opening in iaa7 was restored to wild type with IAA but oxIAA treatment had little effect. [file NIHMS2127192-supplement-Supplementary_Data.pdf]

Jenness et al. Cytosolic- and membrane-localized oxidized auxin formation regulates developmental auxin transients

Supplementary data

Supplementary Table 1. Fluorometric quantitation of DAO1-YFP in soluble and microsomal fractions with and without IAA treatment.
Supplementary Table 2. LC-MS/MS mass transitions and retention times of standards.
Supplementary Table 3. Recombinant DAO1 and DAO2 activity.
Supplementary Table 4. Native IAAsp vs IAA oxidase activity is predominant in soluble fractions and to a lesser extent in membrane fractions.
Supplementary Table 5. Spatio-temporal transcriptome analyses of *DAO1* and *DAO2*.
Supplementary Table 6. Accession numbers
Supplementary Table 7. Lines used in this study
Supplementary Table 8. Primers used for Reverse Transcriptase Real-Time Quantitative PCR.
Supplementary Table 9. Yeast strains used in this study.

Supplementary Figure 1. Construction of *dao2* CRISPR lines and expression in *dao1* mutants.
Supplementary Figure 2. DAO2 is associated with soluble and membrane fractions.
Supplementary Figure 3. Post-translation modification assays indicate N-terminal acetylation mediates DAO1 membrane residence.
Supplementary Figure 4. IAA metabolites in roots and shoots of *dao* alleles grown in continuous light support primary oxidation of IAAsp.
Supplementary Figure 5. Hypocotyl length in 4 d etiolated seedlings.
Supplementary Figure 6. Plant height of *dao* mutants at 43 d.
Supplementary Figure 7. Supernumerary petals in double mutants.
Supplementary Figure 8. DII-Venus signals in etiolated apical hooks are less persistent than DR5 signals.
Supplementary Figure 9. Complemented *dao1* hook and *dao2* hook.
Supplementary Figure 10. IAA levels UH/hooks in etiolated seedlings increase with light.

Supplementary Figure 11. NPA treatment increases IAA/oxIAA levels and relaxes apical hook angles at 12 h.

Supplementary Figure 12. Auxin production in *yuc6-1D* alters developmental timing but not hypocotyl growth rate or light induced hook opening.

Supplementary Figure 13. Protoplast loading of IAA and oxIAA at two different temperatures.

Supplementary Figure 14. Exogenous oxIAA delays hook opening.

Supplementary Figure 15. Apical hook opening in *dao1* is enhanced with IAA and retarded with oxIAA and hook opening in *iaa7* was restored to wild type with IAA but oxIAA treatment had little effect.

Supplementary Table 1. Fluorometric quantitation of DAO1-YFP in soluble and microsomal fractions with and without IAA treatment. The negative (non-specific binding) soluble control is anti-YFP and the positive microsomal control is ABCB4-YFP. Soluble and microsomal fractions were prepared independently from those described in **Fig. 1D**. Three replicates were assayed from each sample and the experiment was repeated in three times. Black protein-binding 96-well plates (Greiner, Kremsmünter Austria) were incubated with a saturating solution of anti-YFP antibody in PBS, washed three times with PBS/ 1% powdered milk, incubated with 25 µg protein (determined by amido black) at 12°C for 1h, then washed three times with PBS-T. YFP fluorescence was assayed on a Synergy HT fluorescence plate reader (485/20 excitation, 530/20 emission, 20 X 2s reads per well). Wells washed with PBS / 1% milk were read as blank and wells incubated with saturating rYFP were used to set arbitrary unit maximum.

Sample	Mean ± SD	Combined Mean ± SD	Percent
Negative control 1	12.33 ± 3.06		
Negative control 2	10.00 ± 2.65	11.22 ± 2.39	100%
Negative control 3	11.33 ± 1.53		
Positive control 1	113.67 ± 1.53		
Positive control 2	111.00 ± 1.00	113.56 ± 2.51	100%
Positive control 3	116.00 ± 1.73		
Soluble solvent only 1	37.33 ± 1.53		
Soluble solvent only 2	32.00 ± 1.73	36.89 ± 4.28	30%
Soluble solvent only 3	41.33 ± 1.53		
Microsomal solvent only 1	89.33 ± 1.16		
Microsomal solvent only 2	86.00 ± 1.00	87.89 ± 1.76	70%
Microsomal solvent only 3	88.33 ± 1.12		
Soluble 100 nM 1	68.33 ± 1.53		
Soluble 100 nM 2	74.00 ± 1.00	70.33 ± 3.08	59%
Soluble 100 nM 3	68.67 ± 2.08		
Microsomal 100 nM 1	49.33 ± 0.58		
Microsomal 100 nM 2	53.67 ± 2.52	49.44 ± 3.97	41%
Microsomal 100 nM 3	45.33 ± 2.08		
Soluble 1 uM 1	63.33 ± 1.53		
Soluble 1 uM 2	66.33 ± 1.15	65.11 ± 2.09	54%
Soluble 1 uM 3	65.67 ± 2.52		
Microsomal 1 uM 1	52.67 ± 1.53		
Microsomal 1 uM 2	56.00 ± 1.73	55.67 ± 3.08	46%
Microsomal 1 uM 3	58.33 ± 2.89		

Supplementary Table 2. LC-MS/MS mass transitions and retention times of standards.

(A) UMD Exposome

Compound	Precursor Ion	Product Ion(s)	Retention Time	Collision Energy	Frag-mentor	Cell Accel	Dwell Time	Standard from
IAIle	289	130	5.25	30	80	7	15, *50*	Olchemim
IAPhe	323	130	5.35	30	95	7	15, *50*	Toronto Research Chemicals
^{13}C-IAA	182	136,81	4.57	12	80	7	15, *50*	Olchemim
IAA	176	130, 77	4.57	12, 48	80	7	15, *50*	Sigma/Olchemim
IAAla	247	130	4.31					Olchemim
oxIAA	192	146	3.91	9, 28	65	7	15	Toronto Research Chemicals, Olchemim
oxIA-Asp	307	146	3.424	26	70	5		Olchemin
IAAsp	291	134,130	3.79	20	80	7	29	Toronto Research Chemicals
IAGlu	305	148	5.25	22	85	7	29	Olchemim
IProA	190	130,103	5.08					Sigma
Methyl IAA	190	103	4.89					Sigma
IATrp	323	130	6.27	30	95			Olchemin

(B) Ohio State Metabolomics

Compound	Precursor Ion	Product Ion(s)	Retention Time	Collision Energy	Frag-mentor	Cell Accel	Dwell Time	Standard from
IATrp-d^5	368	134	8.203	30	95	7	15, *50*	Olchemim
IATrp	362	130	8.100-8.203	30	95	7	15, *50*	Sigma
IAA-d^5	181	134	7.265	12	80	7	15, *50*	Olchemim
IAA	176	130, 77	7.265	12, 48	80	7	15, *50*	Sigma/Olchemim
IAN	157	130	7.539					Olchemim
oxIAA	196	146,128	5.27	9, 28	65	7	15	Sigma/Olchemim
Trp	205	188, 146	3.114	3, 13	70	7	29	Sigma
Trp-d^3	208	191, 147	3.114	3, 13	70	7	29	Olchemim

(C) Settings

MS: Gas temp 250°C, 10L/min.; nebulizer 35 psi, sheath gas 350°C, 12 L/min.; capillary, 2250 + / 2250 –
Chromatography: Pump model 1290 Infinity 2. Measured flow rate 0.300 mL/min. Agilent Poroshell C18 column. Buffer A: 5% MeOH, 0.1% acetic acid; Buffer B: 100% MeOH 0.1% acetic acid. Gradient: 2% buffer B at time 0, brought to 50% at 3 minutes, increased to 98% at 5 minutes, held for 2 minutes, then brought back to 2% at 8 minutes. (Stasko et al., 2020).

Supplementary Table 3. Recombinant DAO1 and DAO2 activity. Metabolites were measured by LC-MS/MS. Assays were conducted as in Hayashi et al., 2021 with similar results, i.e., IAAsp is the preferred substrate of DAO1 and DAO2, except a small amount of oxIAA is formed after 60 min. Metabolites were measured by LC-MS. LOD, limit of detection.

	IAA [nmol per total sample aliquot (100 microL)]									
**Time (min)**	**0**	**5**	**10**	**15**	**20**	**25**	**30**	**40**	**50**	**60**
**DAO1 + IAA**	94.07 ± 0.84	93.88 ± 1.28	93.43 ± 0.33	92.59 ± 0.93	91.97 ± 0.22	91.76 ± 0.29	91.15 ± 0.08	90.89 ± 0.36	90.53 ± 0.36	90.30 ± 0.28
**DAO2 + IAA**	94.08 ± 0.32	94.08 ± 0.56	92.79 ± 0.16	92.82 ± 0.15	92.55 ± 0.15	92.34 ± 0.14	92.09 ± 0.07	91.58 ± 0.29	91.42 ± 0.20	91.22 ± 0.37

	oxIAA [nmol per total sample aliquot (100 microL)]									
**Time (min)**	**0**	**5**	**10**	**15**	**20**	**25**	**30**	**40**	**50**	**60**
**DAO1 + IAA**	LOD	LOD	LOD	LOD	LOD	LOD	2.078 ± 0.50	2.83 ± 0.78	2.82 ± 0.92	3.12 ± 0.72
**DAO2 + IAA**	LOD	LOD	LOD	LOD	LOD	LOD	LOD	1.59 ± 0.13	2.28 ± 0.12	2.87 ± 0.13

	IAAsp [nmol per total sample aliquot (100 microL)]									
**Time (min)**	**0**	**5**	**10**	**15**	**20**	**25**	**30**	**40**	**50**	**60**
**DAO1 + IAAsp**	93.66 ± 1.27	90.42 ± 0.08	69.19 ± 0.21	44.77 ± 0.13	20.55 ± 0.43	6.565 ± 0.47	4.56 ± 0.56	2.92 ± 0.20	1.37 ± 1.03	0.55 ± 0.17
**DAO2 + IAAsp**	93.93 ± 0.88	90.74 ± 0.34	70.76 ± 0.25	46.59 ± 0.21	25.82 ± 0.05	13.35 ± 0.23	11.30 ± 0.47	11.10 ± 0.63	10.73 ± 0.17	9.18 ± 0.49

	oxIAAsp [nmol per total sample aliquot (100 microL)]									
**Time (min)**	**0**	**5**	**10**	**15**	**20**	**25**	**30**	**40**	**50**	**60**
**DAO1 + IAAsp**	LOD	4.16 ± 0.13	26.73 ± 0.26	52.53 ± 0.23	77.98 ± 0.40	92.67 ± 0.61	94.81 ± 0.55	96.61 ± 0.45	97.82 ± 1.05	99.04 ± 0.19
**DAO2 + IAAsp**	LOD	3.97 ± 0.07	25.12 ± 0.19	50.59 ± 0.30	72.44 ± 0.70	85.53 ± 0.30	87.63 ± 0.52	87.94 ± 0.64	88.34 ± 0.15	89.89 ± 0.58

	IAA [nmol per total sample aliquot (100 microL)]									
**Time (min)**	**0**	**5**	**10**	**15**	**20**	**25**	**30**	**40**	**50**	**60**
**DAO1 + IAAsp**	LOD	LOD	LOD	LOD	LOD	LOD	LOD	LOD	LOD	1.43 ± 0.32
**DAO2 + IAAsp**	LOD	LOD	LOD	LOD	LOD	LOD	LOD	LOD	LOD	1.56 ± 0.43

	oxIAA [nmol per total sample aliquot (100 microL)]									
**Time (min)**	**0**	**5**	**10**	**15**	**20**	**25**	**30**	**40**	**50**	**60**
**DAO1 + IAAsp**	LOD	LOD	LOD	LOD	LOD	LOD	LOD	LOD	0.41 ± 0.05	0.41 ± 0.05
**DAO2 + IAAsp**	LOD	LOD	LOD	LOD	LOD	LOD	LOD	LOD	0.32 ± 0.32	0.73 ± 0.73

	Asp [nmol per total sample aliquot (100 microL)]								
**Time (min)**	**0**	**5**	**10**	**15**	**20**	**25**	**30**	**40**	**50**	**60**
**DAO1 + IAAsp**	LOD	LOD	LOD	LOD	LOD	LOD	LOD	LOD	0.51 ± 0.45	1.38 ± 0.53
**DAO2 + IAAsp**	LOD	LOD	LOD	LOD	LOD	LOD	LOD	LOD	LOD	LOD

Supplementary Table 4. LC-MS/MS metabolite analysis shows that native IAAsp oxidase activity predominates in soluble and membrane fractions. (**A**) Solvent (buffer) controls (no substrate added) for assays of metabolic activity of soluble and membrane fractions from Col-0, *dao1-1*, *dao1-1dao2-4*, and *irl1ill2iar3.* In solvent controls, only oxIAA was detectable. The microsomal fractions were washed twice. LOD, limit of detection.
(**B**) Oxidative activity of soluble fractions from Col-0, *dao1-1*, *dao1-1dao2-4*, and *irl1ill2iar3* after addition of IAA or IAAsp. Results are consistent with primary oxidative activity of DAO1 and DAO2 with IAAsp and very low activity against IAA. "-", IAAsp was not measured in assays where IAAsp was added. The microsomal fractions were washed twice. **(C)** Oxidation activity of microsomal membranes from Col-0, *dao1-1*, *dao1-1dao2-4*, and *irl1ill2iar3* indicates IAAsp oxidation is primary activity of membrane-associated DAO, but that oxidation of IAA is slightly increased compared to soluble fraction. Add back of soluble fraction from double amidohydrolase mutant increases production of oxIAAsp and not oxIAA from both IAA and IAAsp. Note: observed conversion of substrates in Col-0 averaged ~15% for IAAsp and 8% for IAA. A limitation of these assays is that only 9-14% of the original IAA substrate was converted in 30 minutes when ATP was no longer detectable. The near neutral pH of the assay buffer was used to limit IAA uptake by membranes and to optimize DAO activity, but would also be expected to reduce GH3 enzymatic activity which exhibit optima at pH 8 (LeClere et al., 2002). "-", IAAsp was not measured in assays where IAAsp was added. LOD, limit of detection.

(A)

	pmol mg^{-1} protein		
**Buffer controls**	**IAAsp**	**oxIAAsp**	**oxIAA**
Soluble fractions			
Col-0	LOD	LOD	11.18 ± 2.47
dao1	LOD	LOD	LOD
dao1dao2	LOD	LOD	LOD
Microsomal fractions			
Col-0	LOD	LOD	LOD
dao1	LOD	LOD	LOD
dao1dao2	LOD	LOD	LOD
dao1 + *ilr1ill2iar3* soluble	LOD	0.08 ± 0.02	0.73 ± 0.24
dao1dao2 + *ilr1ill2iar3* soluble	LOD	LOD	LOD

(B)

	pmol mg^{-1} protein		
Soluble fractions	**IAAsp**	**oxIAAsp**	**oxIAA**
Col-0 + IAA	70.86 ± 5.09	10.83 ± 1.29	105.21 ± 4.65
Col-0 + IAAsp	-	93.11 ± 6.79	112.05 ± 3.88
dao1 + IAA	19.5 ± 3..32	2.11 ± 1.04	9.54 ± 0.31
dao1 + IAAsp	-	8.2 ± 3.11	16.65 ± 0.00
dao1dao2 + IAA	98.76 ± 15.11	1.19 ± 0.16	13.48 ± 2.17
dao1dao2 + IAAsp	-	12.51 ± 6.06	23.43 ± 0.00

(C)

	pmol mg^{-1} protein		
Microsomal fractions	**IAAsp**	**oxIAAsp**	**oxIAA**
Col-0 + IAA	LOD	0.91 ± 0.00	6.45 ± 0.00
Col-0 + IAAsp	-	15.41 ± 0.00	0.17 ± 0.00
dao1 + IAA	LOD	0.11 ± 0.06	LOD
dao1 + IAAsp	-	0.64 ± 0.05	LOD
dao1dao2 + IAA	LOD	LOD	LOD
dao1dao2 + IAAsp	-	0.43 ± 0.01	LOD
dao1dao2 + *ilr1ill2iar3* soluble + IAA	1.80 ± 0.29	6.73 ± 2.53	LOD
dao1dao2 + *ilr1ill2iar3* soluble + IAAsp	-	16.49 ± 2.91	LOD

Supplementary Table 5. **Spatio-temporal transcriptome analyses of *DAO1* and *DAO2*.**

(http://travadb.org/)

Tissue	*DAO1*	*DAO2*
Dry seeds	425	22
Germinating seeds (first day after soaking)	165	14
Germinating seeds (second day after soaking)	165	708
Germinating seeds (third day after soaking)	218	323
Seedling meristem	187	57
Seedling cotyledons	138	67
Seedling hypocotyl	279	821
Seedling root	111	6
Shoot Apical Meristem (SAM)	288	12
Root without apex	263	515
Root apex	148	18
SAM at 8 DAG	177	3
SAM at 9 DAG	238	0
Meristem at 10 DAG	211	0
Meristem at 11 DAG	288	0
Meristem at 12 DAG	215	1
Inflorescence meristem at 13 DAG	201	0
Inflorescence meristem at 14 DAG	215	0
Inflorescence meristem at 15 DAG	236	8
Inflorescence meristem at 16 DAG	230	19
Petiole of the young leaf	114	1
Leaf blade of the young leaf	113	0.54
Petiole, intermediate 1	125	26
Leaf blade, intermediate 1	119	2
Petiole, intermediate 2	155	34
Leaf blade, intermediate 2	166	1
Leaf vein, intermediate 2	125	0
Petiole of the mature leaf	261	3

Leaf blade of the mature leaf	206	0.53
Vein of the mature leaf	216	2
Whole mature leaf	220	3
Pedicel	129	0.36
Axis of the inflorescence	146	0
Internode	292	0
Flower 1	416	0.45
Flower 2	315	0
Flower 3	319	5
Flower 4	328	11
Flower 5	176	3
Flower 6-8	170	2
Flower 9-11	199	9
Flower 12-14	158	6
Flower 15-18	154	1
Flower 19+	175	4
Sepals of the young flower	173	0
Anthers of the young flower	147	0
Carpels of the young flower	239	24
Ovules from 6th and 7th flowers	336	2
Stigmatic tissue	412	26
Carpel of 6th and 7th flowers	212	2
Sepals of the mature flower	672	0
Petals of the mature flower	202	0
Stamen filaments of the mature flower	282	0.98
Anthers of the mature flower (before opening)	708	9
Carpels of the mature flower (before pollination)	252	0.46
Opened anthers	331	0
Young seeds 1	471	0
Young seeds 2	501	0

Young seeds 3	660	0
Young seeds 4	688	0
Young seeds 5	569	0
Seeds 1	318	5
Seeds 3	680	0.47
Seeds 5	406	4
Seeds 7	473	1
Pod of the silique 1	513	3
Pod of the silique 3	363	2
Pod of the silique 5	345	0.59
Pod of the silique 7	304	0
Silique 2	440	5
Silique 4	417	2
Silique 6	437	0.55
Silique 8	326	0.53
Petiole of the senescent leaf	752	9
Vein of the senescent leaf	813	2
Seeds of first yellowing silique	605	15
Pod of the senescent silique 1	9250	14
Senescent silique 2	1401	8
Senescent internode	314	2

Supplementary Table 6. Accession numbers

Gene	Gene Locus
DIOXYGENASE FOR AUXIN OXIDATION 1 (*DAO1*)	AT1G14130
DIOXYGENASE FOR AUXIN OXIDATION 2 (*DAO2*)	AT1G14120
GRETHEN HAGEN 3.3 1AA-amido synthetase (*GH3.3*)	AT2G23170
PLASMA MEMBRANE INTRINSIC PROTEIN 2A (*PIP2A*)	AT3G53420
VACUOLAR PROTON ATPase subunit A3 (*VHA-A3*)	AT4G39080
RAB GTPase RAB A2A (*RABA2A*)	AT1G09630
SAR1 guanine nucleotide exchange factor 12 (*SEC12*)	AT2G01470
YUCCA6 (YUC6)	AT5G25620
IAA-LUECINE RESISTANT 1 (ILR1)	AT3G02875
IAA-LUECINE RESISTANT 1 (ILR1)-LIKE 2 (ILL2)	AT5G56660
IAA-ALANINE RESISTANT 3 (IAR3)	AT1G51760
INDOLE-3-ACETIC ACID 7/AUXIN RESISTANT 2 (IAA7/AXR2)	AT3G23050
INDOLE-3-ACETIC ACID 17/ AUXIN RESISTANT 3 (IAA17/AXR3)	AT1G04250
TRANSMEMBRANE KINASE 1 (TMK1)	AT1G66150

Supplementary Table 7. Lines used in this study

Line	Reference
dao1-1	Zhang *et al.*, 2016
dao2-2	This study
dao2-3	This study
dao1-1 dao2-4	This study
dao1-1 dao2-5	This study
ilr1 ill2 iar3	Rampey et al., 2004
*DAO1*pro:GUS	Zhang *et al.*, 2016
*DAO2*pro:GUS	This study
DR5:GUS	Ulmasov *et al.*, 1997
DR5rev:GFP	Friml *et al.*, 2003
axr2-1	Timpte *et al.*, 1994
iaa7-7	Reed *et al.*, 2018
iaa17-7	Reed *et al.*, 2018
tmk1-1	Cao *et al.*, 2019; Lin *et al.*, 2021
yuc6-1D	Kim *et al.*, 2007

Supplementary Table 8. Primers used for Reverse Transcriptase Real-Time Quantitative PCR (RT-qPCR) and sequencing.

Primer	Sequence	Purpose
DAO2_Seq_F1	ATGGCGGAAGTAAATGGAGTC	Sequencing
DAO2_Seq_F2	CGGAAGTAAATGGAGTCATTCCAAC	Sequencing
DAO2_Pro_F	CAGACTTCTGTGCATGGTCGA	Amplifying region flanking the guideRNA site
DAO2_525_R	GCTAGACTCCTCGCAAGATCATC	Amplifying region flanking the guideRNA site
DAO1-F	ATCCGTTGCAAGTCCATTGA	RT-qPCR
DAO1-R	GTTACAGAGCTCCAAACGAAA	RT-qPCR
DAO2-F	AAAATTGGGCTCTACCACTCC	RT-qPCR
DAO2-R	TTGTGATAAACTCGACGCCTC	RT-qPCR
UBQ5-F	GACGCTTCATCTCGTCC	RT-qPCR
UBQ5-R	CCACAGGTT GCGTTAG	RT-qPCR
GH3.3-F	ACAATTCCGCTCCACAGTTC	RT-qPCR
GH3.3-R	ACGAGTTCCTTGCTCTCCAA	RT-qPCR
SUR2-F	CGGTTAGAGAAGAAGAGT	RT-qPCR
SUR2-R	TACATACGACACAGTTAGT	RT-qPCR

Supplementary Table 9. Yeast strains used in this study

Name	Plasmids	Auxotrophy
YFP-Stop	pGP4GY-Stop	-Trp
5G-TIR1|YFP-IAA7	pGP5G-TIR1, pGP4GY-IAA7	-Trp-Leu
5G-TIR1|YFP-IAA17	pGP5G-TIR1, pGP4GY-IAA17	-Trp-Leu

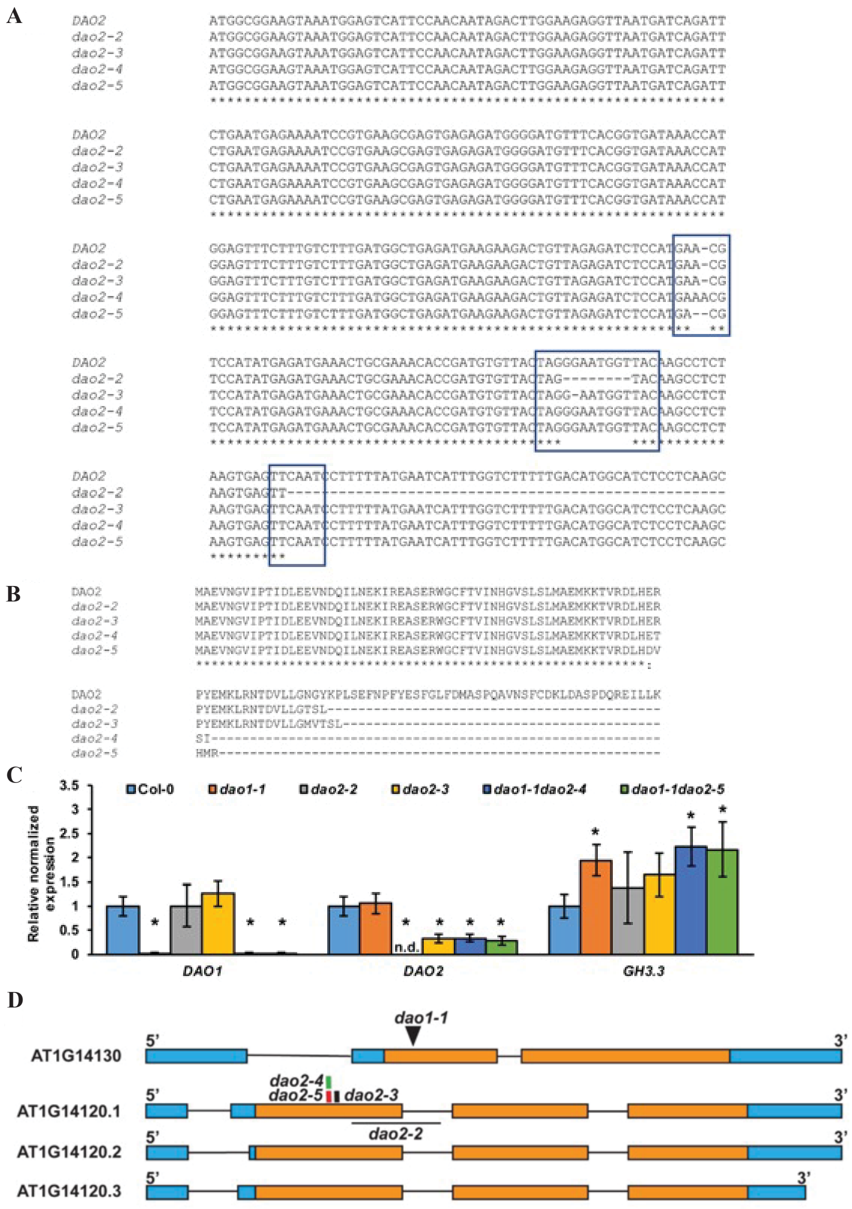

Supplementary Figure 1. Construction of *dao2* CRISPR lines and expression in *dao1* mutants.

(A-B) DNA (**A**) and protein (**B**) sequence alignment of *dao2* alleles indicating position of CRISPR-Cas9 inducted deletions and insertions. *DAO1* and *DAO2* are separated by 1207 bp. **C.** Reverse Transcriptase Real-Time Quantitative PCR of *DAO1*, *DAO2*, and *GH3.3* in *dao* single and double mutants. Data shown are means ± SD (n=3 biological replicates, 2 technical replicates). n.d.: not detected. Asterisks indicate statistical difference from Col-0 by Student's *t*-

test (P < 0.05). **D.** Gene models of *DAO2* and *DAO2* and locations of mutations (araport.org). The blue rectangles represent untranslated regions of the genes. The orange rectangles reparent translated regions of the genes. The black triangle represents a T-DNA insertion. The red, green and black rectangles represent the CRISPR-Cas9 mutation sites. The line about *dao2-2* represents the deleted nucleotides.

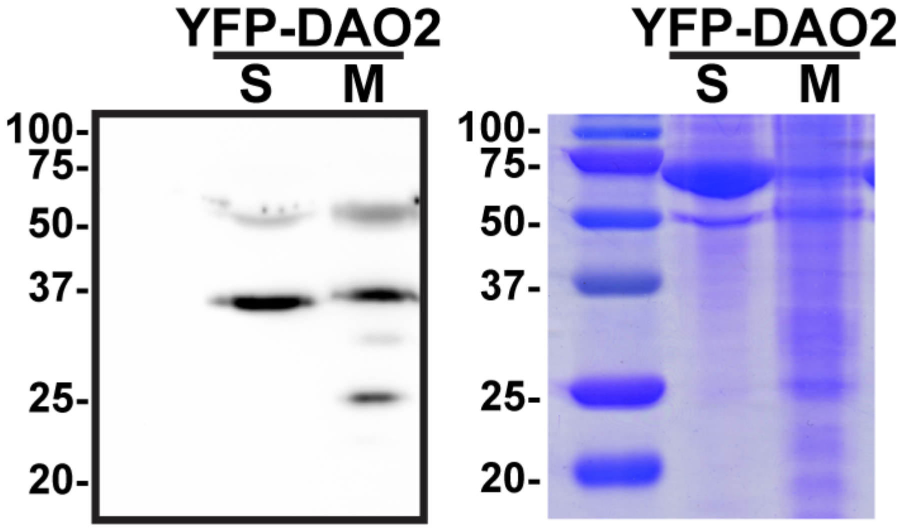

Supplementary Figure 2. Western blot analysis of YFP-DAO2. S, soluble fraction; M, microsomal fraction. Western blot is on the left. CCB stained gel is on the right.

A

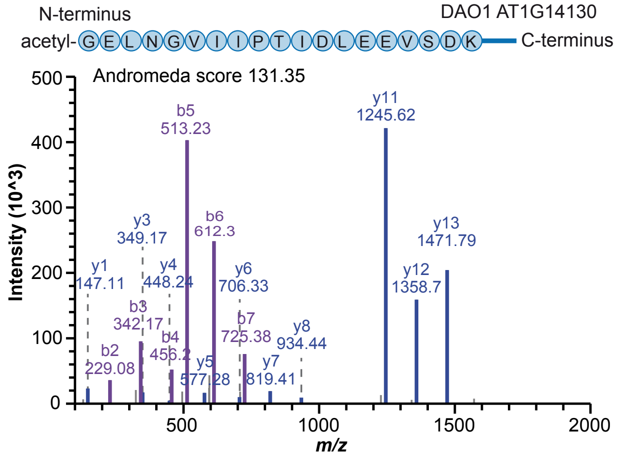

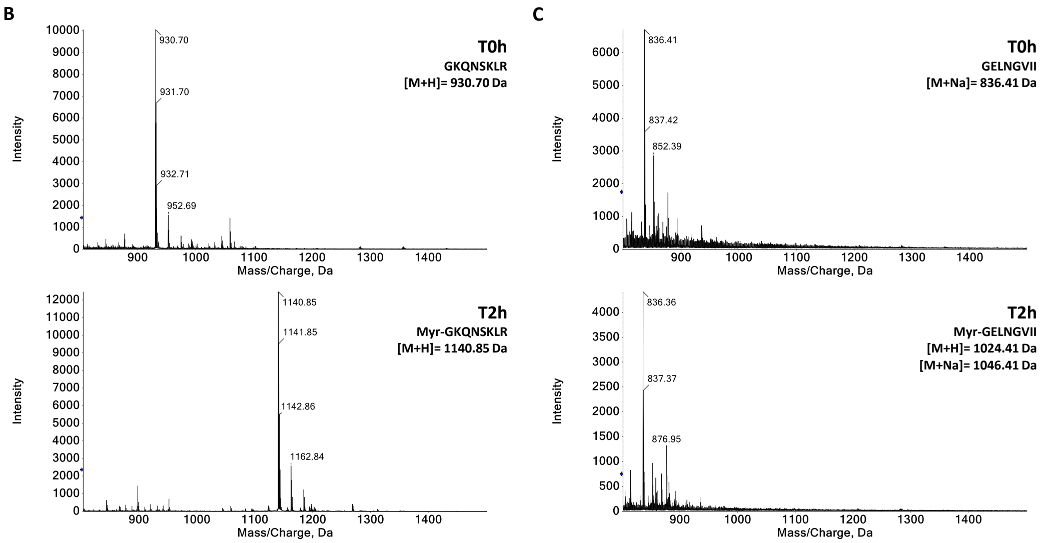

D

>AT1G14130.1 | 2-oxoglutarate (2OG) and Fe(II)-dependent oxygenase superfamily protein | Chr1:4836041-4837040 REVERSE LENGTH=308
MGELNGVIIPTIDLEEVSDKILNQKIREASERWGCFRVINHGVSLSLMAEMKKTVIDLFQRPYEVKVRNTDVLLGSGYRAPNEINPYYEALGLYDMAS
PHAVNTFCDQLEASADQREIMVKYAKAINGLATDLARKLAESYGLVETDFFKEWPSQFRINKYHFKPETVGKLGVQLHTDSGFLTILQDDENVGGLE
AMDNSSGTFFPIDPLPNTLAINLGDMATIWSNGRLCNVKHRVQCKEATMRYSIASFLLGPMDTDLEPPSEFVDAEHPRLYKPISHEGVRNIRMTKKL
HDGEALKLITHAGLDK

>AT1G14120.1 | 2-oxoglutarate (2OG) and Fe(II)-dependent oxygenase superfamily protein | Chr1:4833648-4834833 REVERSE LENGTH=312
MAEVNGVIPTIDLEEVNDQILNEKIREASERWGCFTVINHGVSLSLMAEMKKTVRDLHERPYEMKLRNTDVLLGNGYKPLSEFNPFYESFGLFDMAS
PQAVNSFCDKLDASPDQREILLKYAKATDDLARSLARRLAESYGVVEPNFLRGWPSQFRMNKYHFKPDSVGKLGVILHTDPGFLTILQGDEDVGGL
EAMDNSSGSFFPIHTLPNTLLVNLGDMATIWSNGRLCNVKHRVQCIEAKMRITIASFLLGPVDRDLEAPDEFVDAEHPRLYKPISDGGLRKIRLSKHLH
AGESLKFITINELSDVPKID

Supplementary Figure 3. Post-translation modification assays indicate N-terminal acetylation mediates DAO1 membrane residence.

(A) N-terminal acetylation of DAO1. Schematic presentation and annotated MS/MS spectra for the N-terminally acetylated peptide of DAO1. Y-ion series (blue), b-ion series (purple). (**B,C**) DAO1 peptide GELNGVII is not myristoylated. MALDI-ToF analysis of a mixture containing

0.5 μM of Arabidopsis NMT1 and 100 μM of synthetic peptide before (T0) and after 2 hours (T2) of reaction at 37°C. **(B)** Positive control (GKQNSKLR), at time 0 (up) or after 2 h of reaction (bottom). The peak corresponding to the non-myristoylated peptide disappeared and a new peak corresponding to the myristoylated peptide appeared (bottom). **(C)** GELNGVII DAO1 peptide, putative myristoylation at 0 h (up) or after 2 h of reaction (bottom). (**D)** Proteomic sequence coverage for DAO1 (At1g14130) and DAO2 (At1g14120). Peptides identified by LC-MS/MS analysis are underlined.

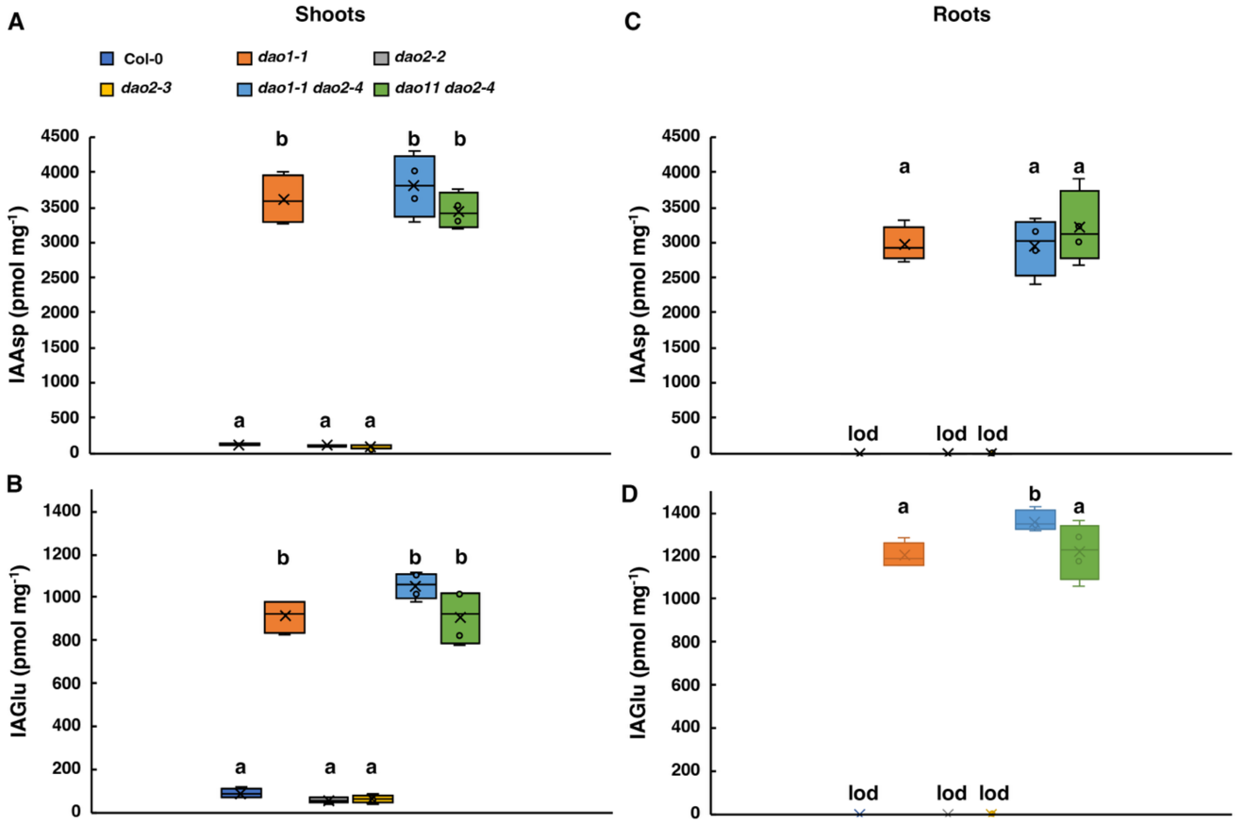

Supplementary Figure 4. IAA-metabolites in roots and shoots of *dao* alleles grown in continuous light support primary oxidation of IAAsp.

IAAsp (**A,C**) and IAGlu (**B,D**) were analyzed in shoots (**A,B**) and roots (**C,D**) of 7-day-old seedlings. Data shown are box plots with whiskers, (n = 5 biological replicates). Center line, median; box limits, upper and lower quartiles; whiskers, 1.5x interquartile range; points, outliers. Letters indicate statistical differences by ANOVA ($P<0.001$) followed by Tukey's post-hoc analysis ($P < 0.05$). "lod", limit of detection.

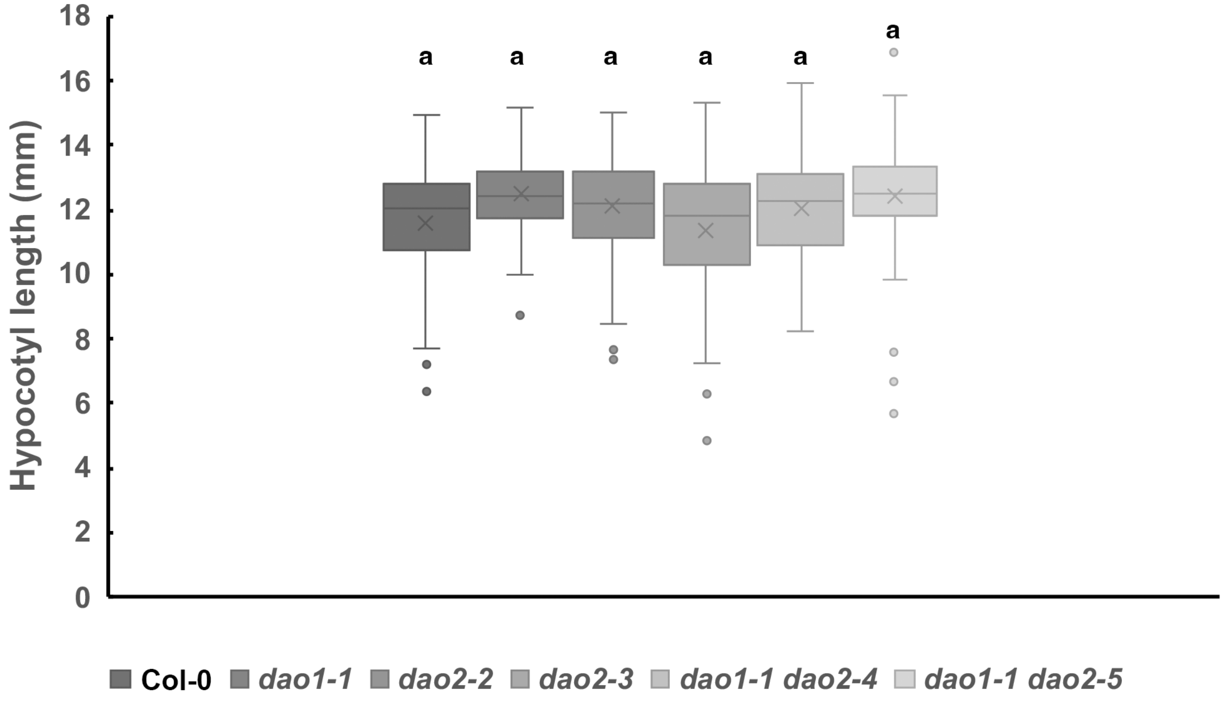

Supplementary Figure 5. Hypocotyl length in 4 d etiolated seedlings. Data shown are box plots with whiskers (n=52-77). Center line, median; box limits, upper and lower quartiles; whiskers, 1.5x interquartile range; points, outliers. Letters indicate statistical differences. No statistical differences by ANOVA and Tukey's post-hoc analysis ($P < 0.05$).

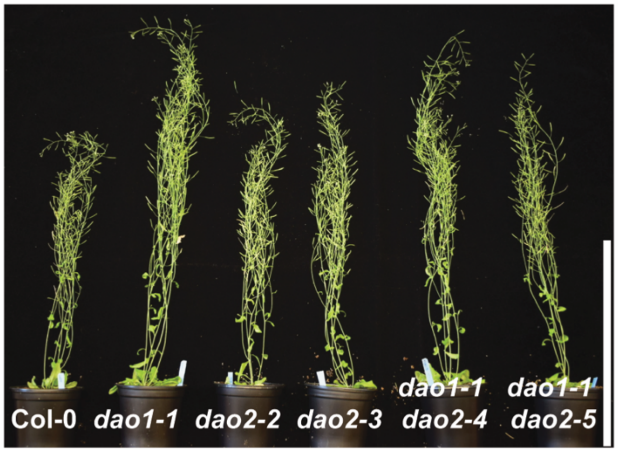

Supplementary Figure 6. Plant height of *dao* mutants at 43 d.

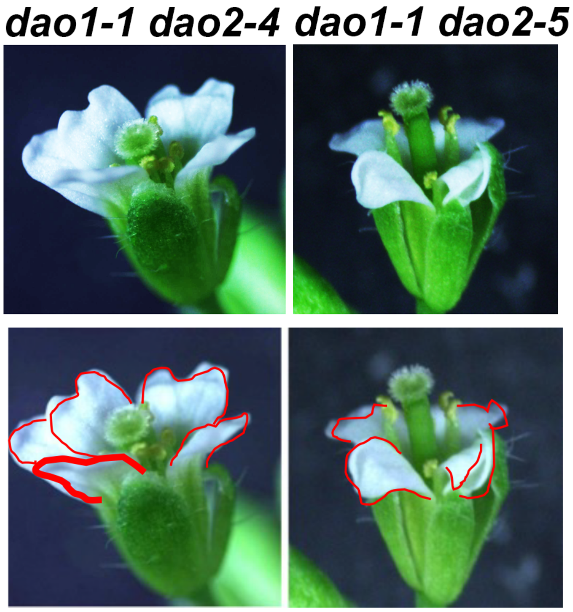

Supplementary Figure 7. Incidence of supernumerary petals in double mutants increases with temperature elevation. Red lines outline petals.

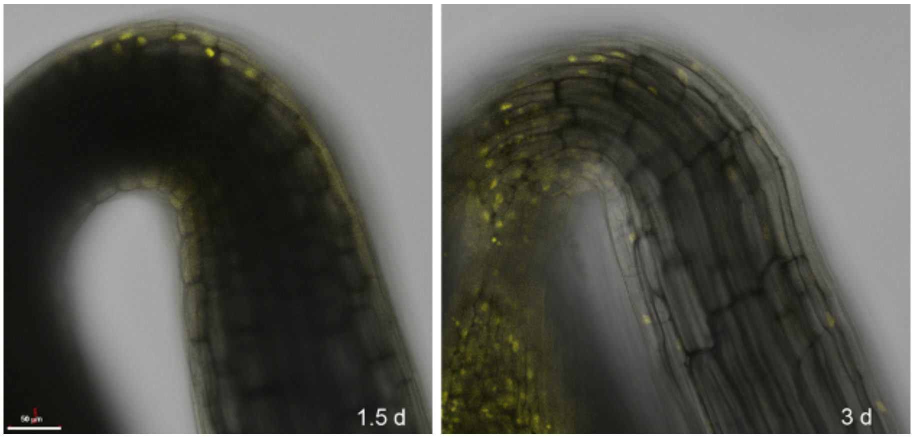

Supplementary Figure 8. DII-Venus signals in etiolated apical hooks are less persistent than DR5-GFP or YFP signals.

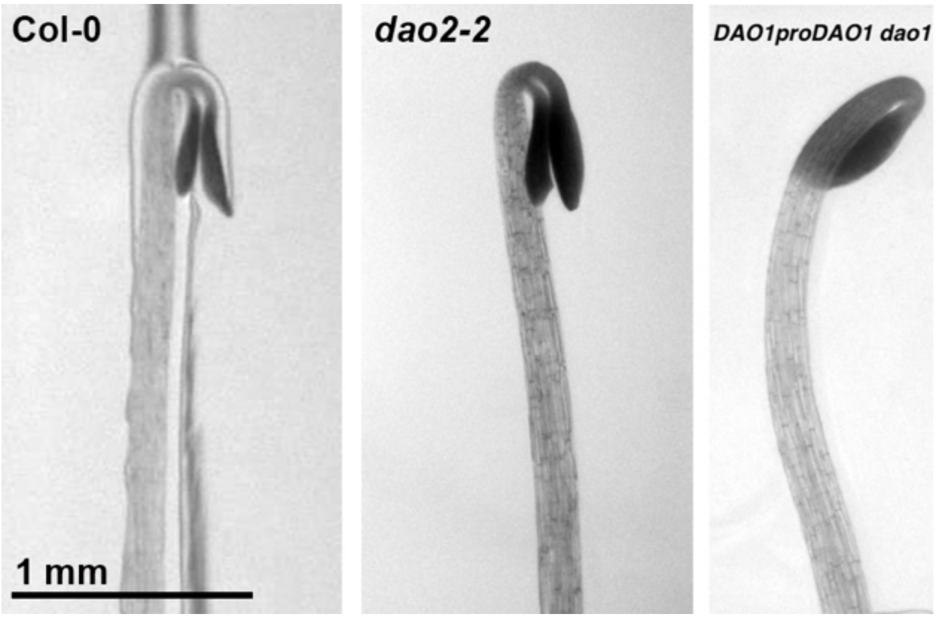

Supplementary Figure 9. Apical hooks in etiolated Col-0, dao2-2 and complemented *dao1-1* (*DAO1pro:DAO1* in *dao1-1*). There is no statistical difference in hook angles at 60 and 72h.

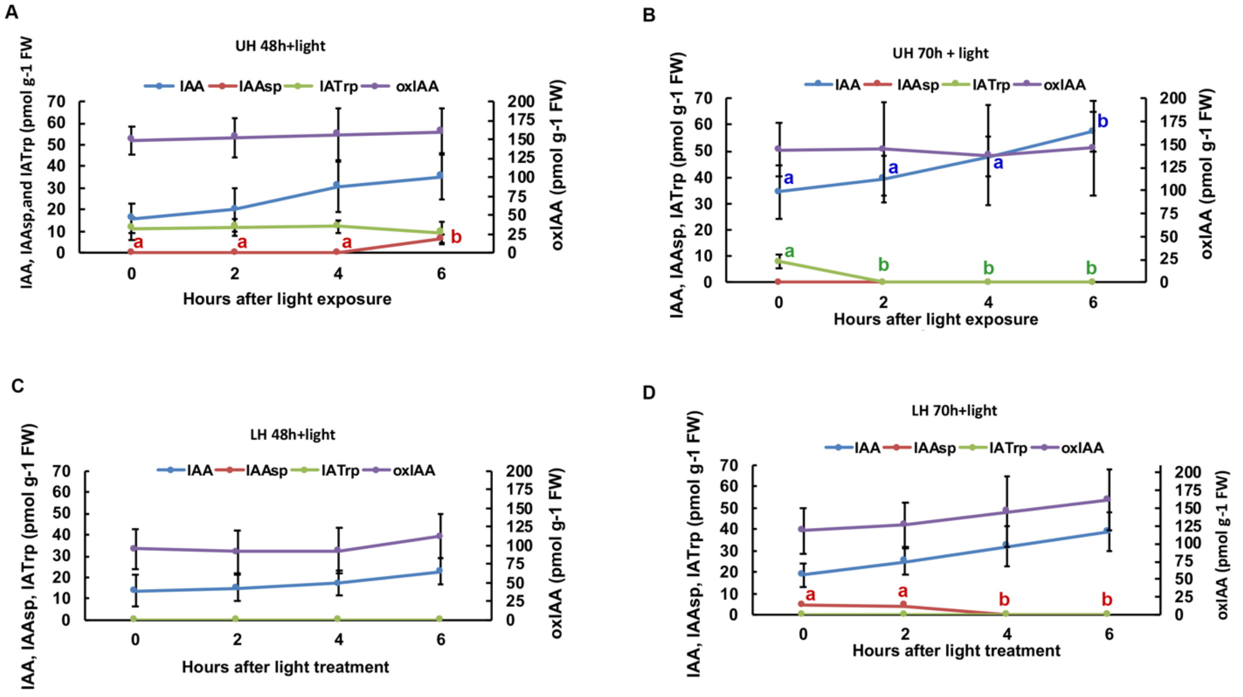

Supplementary Figure 10. IAA levels in etiolated UH/hooks increase with light. Etiolated seedlings were exposed to light at either 48 h **(A, C)** or 70 h **(B, D)** and tissues were harvested over 6 h for auxin metabolite quantitation. **(A-B)** Upper hypocotyl (UH) includes cotyledons, hook, and apical hypocotyl region at the point equivalent to where the cotyledon tips end. **(C-D)** Lower hypocotyl (LH) tissue included the hypocotyl region after dissection at the equivalent to where the cotyledon tips end to the top of the root-shoot transition zone. Data shown are means ± SD (n=5). Letters in colors corresponding to compounds indicate statistical differences over the timecourse by ANOVA followed by Tukey's post-hoc analysis ($P < 0.05$). For clarity, letters are omitted for compounds without statistical differences over the timecourse. Data points on x-axis are at the limit of detection.

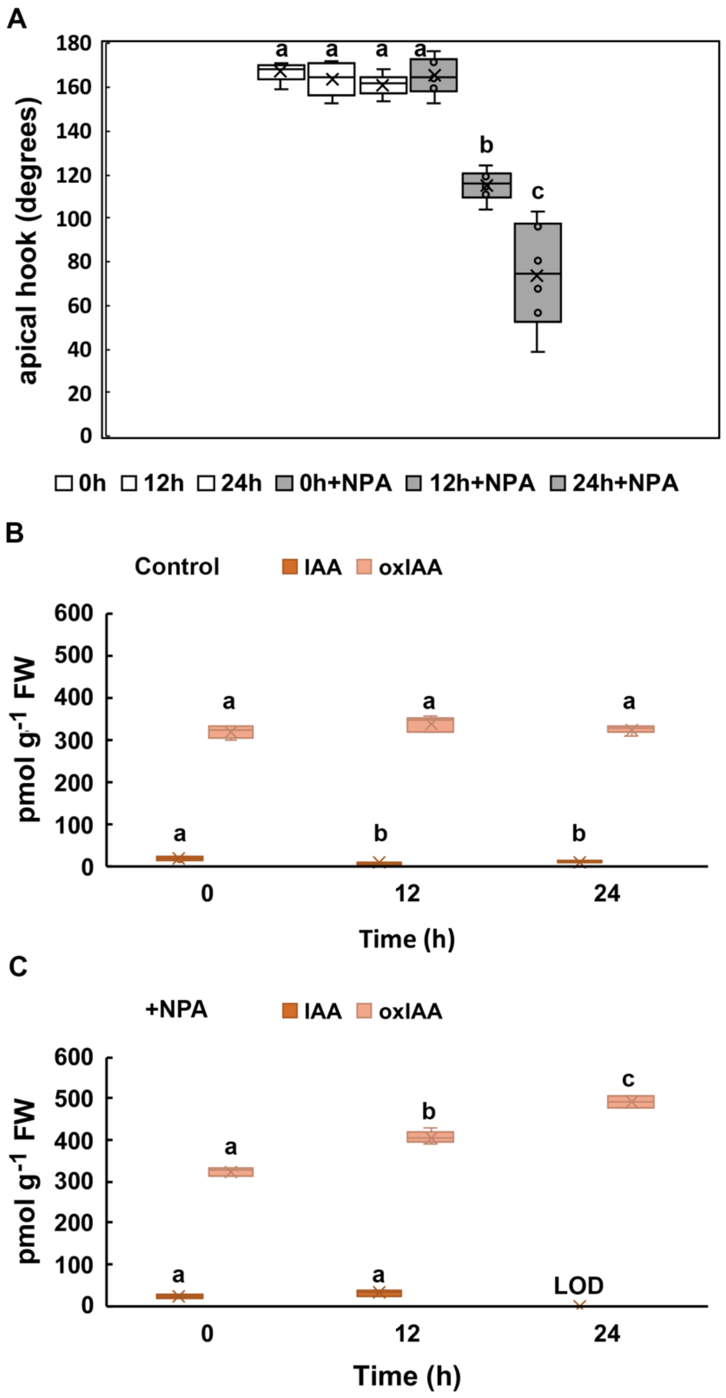

Supplementary Figure 11. NPA treatment increases IAA and oxIAA levels and relaxes apical hook angles at 12 h. Application of 5 µM NPA in lanolin to apical hook at 2 d and then analyzed over 24 h timecourse. (**A**) Apical hook angle. (**B-C**) IAA and oxIAA quantitations. **(B)** Control. **(C)** NPA treatment. LOD, limit of detection. Letters indicated statistical difference by ANOVA followed by Tukey's post-hoc analysis ($P < 0.05$) for each treatment. Data shown are

box plots with whiskers. Center line, median; box limits, upper and lower quartiles; whiskers, 1.5x interquartile range; points, outliers.

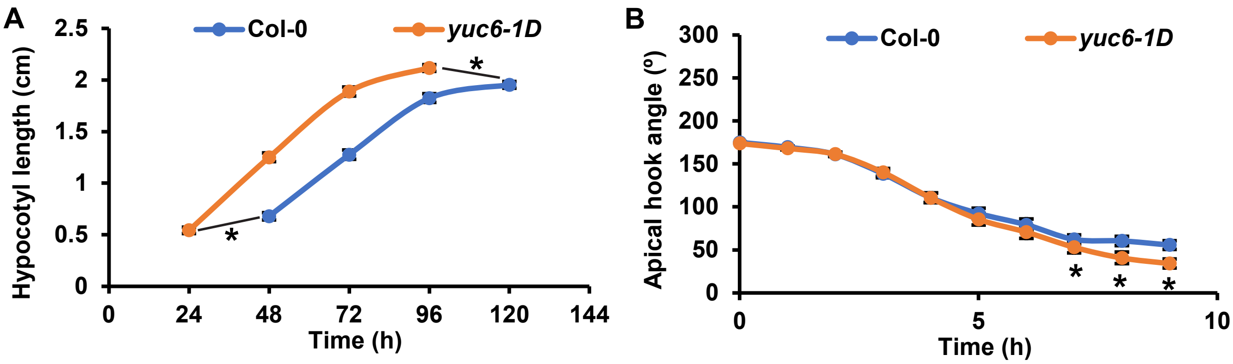

Supplementary Figure 12. Auxin production in *yuc6-1D* alters developmental timing but not hypocotyl growth rate or light induced hook opening.

A Kinetics of hypocotyl elongation in Col-0 and *yuc6-1D*. Data shown are means ± SE (n=15). Asterisks indicate statistical difference between Col-0 and *yuc6-1D* timepoints offset by 24 h by two-tailed Student's t-test (P<0.05). **B** Kinetics of light-induced apical hook opening in Col-0 and *yuc6-1D*. Seedlings were grown in the dark for 48 h and 24 h for Col-0 and *yuc6-1D*, respectively, to equivalent hypocotyl lengths and apical hook angles. Seedlings were then shifted to continuous 100 µmol m^{-2} s^{-1} white light to induce hook opening. Data shown are means ± SE (n=15). Asterisks indicate statistical difference from Col-0 by Kruskal-Wallis (P<0.001) followed by Steels's post-hoc analysis (P<0.05).

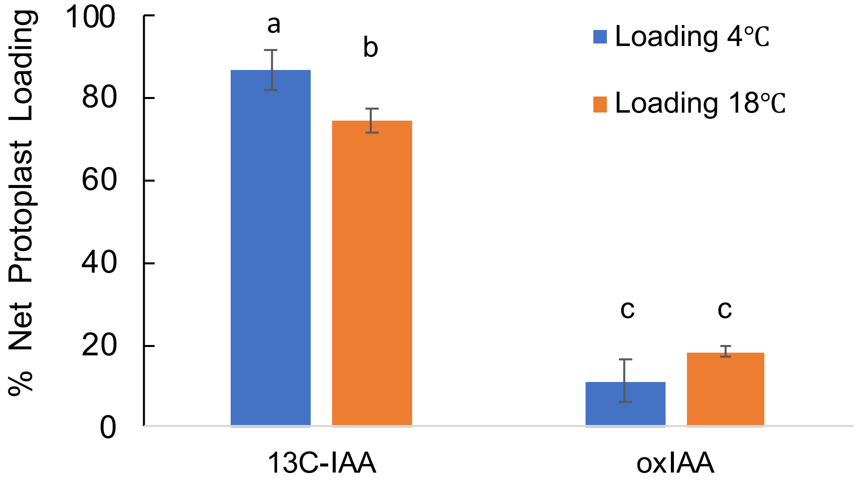

Supplementary Figure 13. Protoplast loading of IAA and oxIAA at two different temperatures. Assay as described in Geisler et al. (2005). Loading as described in Jenness et al. (2020). 50 pmol added per sample. Preloading solvent control mean ± SD for oxIAA 5.47 ± 0.54 at 4° C and 6.34 ± 0.08 at 18° C. Data presented are means ± SD. Letters represent statistical differences. ANOVA $P < 0.001$, Tukey's post-hoc $P < 0.05$.

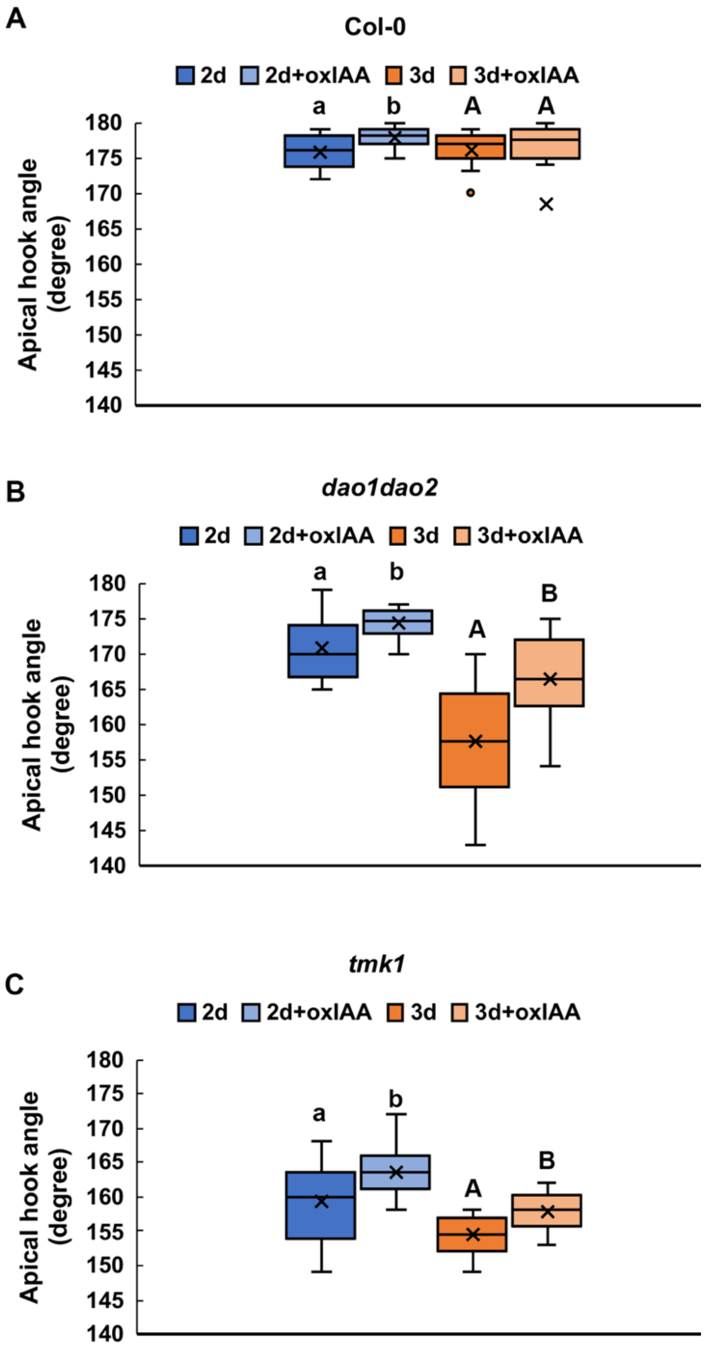

Supplementary Figure 14. Exogenous oxIAA delays hook opening. 5 µM oxIAA in lanolin paste was applied to the concave side of the apical hook of 2- or 3-day-old etiolated (**A**) Col-0, (**B**) *dao1-1dao2-4* and (**C**) *tmk1-1* seedlings. Letters indicated statistical difference by ANOVA followed by Tukey's post-hoc analysis, $P < 0.05$. Data shown are box plots with whiskers. Center line, median; box limits, upper and lower quartiles; whiskers, 1.5x interquartile range; points, outliers.

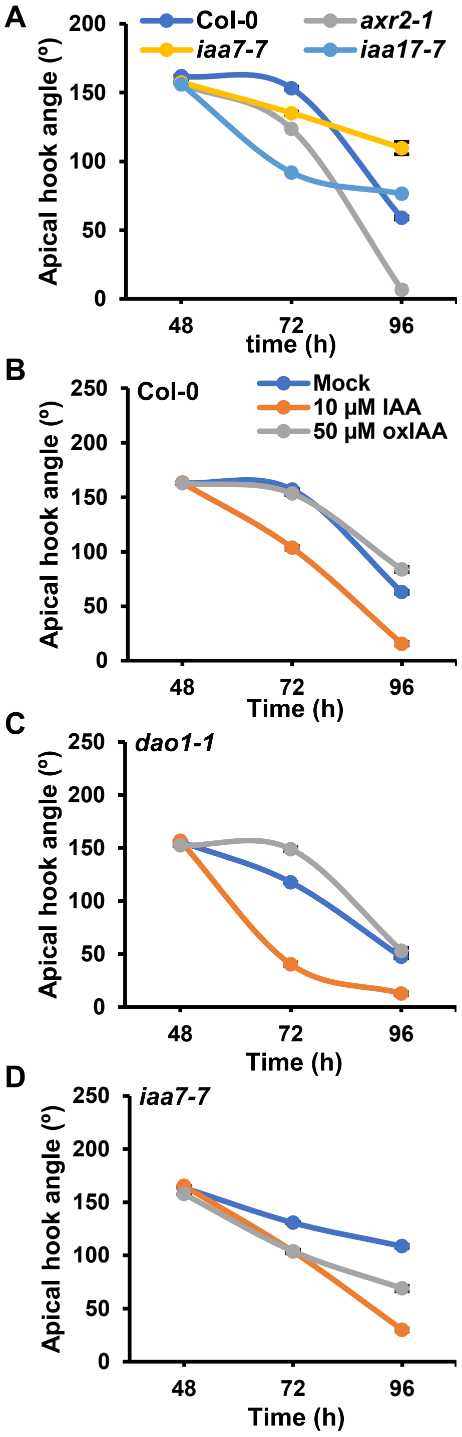

Supplementary Figure 15. Apical hook opening in *dao1* is enhanced with IAA and retarded with oxIAA and hook opening in *iaa7* was restored to wild type with IAA but oxIAA treatment had little effect.

(A) Kinetics of apical hook maintenance and opening in Col-0, *axr2-1*, *iaa7*, and *iaa17*. Data shown are means ± SE (n=10). **(B-D)** Kinetics of apical hook opening in **(B)** Col-0, **(C)** *dao1*, and **(D)** *iaa7* with IAA and oxIAA application. Agarose beads soaked in 10 µM IAA or 50 µM oxIAA were applied to the inner hook region of 48 h seedlings. Data shown are means ± SE (n=15).

Literature cited

Friml, J., Vieten, A., Sauer, M., Weijers, D., Schwarz, H., Hamann, T., Offringa, R., & Jürgens, G. (2003). Efflux-dependent auxin gradients establish the apical-basal axis of Arabidopsis. *Nature*, *426*(6963), 147–153. https://doi.org/10.1038/nature02085

Geisler, M., Blakeslee, J. J., Bouchard, R., Lee, O. R., Vincenzetti, V., Bandyopadhyay, A., Titapiwatanakun, B., Peer, W. A., Bailly, A., Richards, E. L., Ejendal, K. F., Smith, A. P., Baroux, C., Grossniklaus, U., Müller, A., Hrycyna, C. A., Dudler, R., Murphy, A. S., & Martinoia, E. (2005). Cellular efflux of auxin catalyzed by the Arabidopsis MDR/PGP transporter AtPGP1. *The Plant journal : for cell and molecular biology*, *44*(2), 179–194. https://doi.org/10.1111/j.1365-313X.2005.02519.x

Jenness, M. K., Tayengwa, R., & Murphy, A. S. (2020). An ATP-Binding Cassette Transporter, ABCB19, Regulates Leaf Position and Morphology during Phototropin1-Mediated Blue Light Responses. *Plant physiology*, *184*(3), 1601–1612. https://doi.org/10.1104/pp.20.00223

Kim, J. I., Sharkhuu, A., Jin, J. B., Li, P., Jeong, J. C., Baek, D., Lee, S. Y., Blakeslee, J. J., Murphy, A. S., Bohnert, H. J., Hasegawa, P. M., Yun, D. J., & Bressan, R. A. (2007). yucca6, a dominant mutation in Arabidopsis, affects auxin accumulation and auxin-related phenotypes. *Plant physiology*, *145*(3), 722–735. https://doi.org/10.1104/pp.107.104935

Lin, W., Zhou, X., Tang, W., Takahashi, K., Pan, X., Dai, J., Ren, H., Zhu, X., Pan, S., Zheng, H., Gray, W. M., Xu, T., Kinoshita, T., & Yang, Z. (2021). TMK-based cell-surface auxin signalling activates cell-wall acidification. *Nature*, *599*(7884), 278–282. https://doi.org/10.1038/s41586-021-03976-4

Rampey, R. A., LeClere, S., Kowalczyk, M., Ljung, K., Sandberg, G., & Bartel, B. (2004). A family of auxin-conjugate hydrolases that contributes to free indole-3-acetic acid levels during

Arabidopsis germination. *Plant physiology*, *135*(2), 978–988. https://doi.org/10.1104/pp.104.039677

Reed, J. W., Wu, M. F., Reeves, P. H., Hodgens, C., Yadav, V., Hayes, S., & Pierik, R. (2018). Three Auxin Response Factors Promote Hypocotyl Elongation. *Plant physiology*, *178*(2), 864–875. https://doi.org/10.1104/pp.18.00718

Stasko, A. K., Batnini, A., Bolanos-Carriel, C., Lin, J. E., Lin, Y., Blakeslee, J. J., & Dorrance, A. E. (2020). Auxin Profiling and *GmPIN* Expression in *Phytophthora sojae*-Soybean Root Interactions. *Phytopathology*, *110*(12), 1988–2002. https://doi.org/10.1094/PHYTO-02-20-0046-R

Timpte, C., Wilson, A. K., & Estelle, M. (1994). The axr2-1 mutation of Arabidopsis thaliana is a gain-of-function mutation that disrupts an early step in auxin response. *Genetics*, *138*(4), 1239–1249. https://doi.org/10.1093/genetics/138.4.1239

Ulmasov, T., Murfett, J., Hagen, G., & Guilfoyle, T. J. (1997). Aux/IAA proteins repress expression of reporter genes containing natural and highly active synthetic auxin response elements. *The Plant cell*, *9*(11), 1963–1971. https://doi.org/10.1105/tpc.9.11.1963

Zhang, J., Lin, J. E., Harris, C., Campos Mastrotti Pereira, F., Wu, F., Blakeslee, J. J., & Peer, W. A. (2016). DAO1 catalyzes temporal and tissue-specific oxidative inactivation of auxin in Arabidopsis thaliana. *Proceedings of the National Academy of Sciences of the United States of America*, *113*(39), 11010–11015. https://doi.org/10.1073/pnas.1604769113
